# Synthesis and Chemical Properties of 3-Phosphono-coumarins and 1,2-Benzoxaphosphorins as Precursors for Bioactive Compounds

**DOI:** 10.3390/molecules24112030

**Published:** 2019-05-28

**Authors:** Ana I. Koleva, Nevena I. Petkova-Yankova, Rositca D. Nikolova

**Affiliations:** Department of Organic Chemistry and Pharmacognosy, Faculty of Chemistry and Pharmacy, Sofia University “St. Kliment Ohridski”, 1 J. Bouchier Buld., 1164 Sofia, Bulgaria; koleva.ana@gmail.com (A.I.K.); nipetkova@chem.uni-sofia.bg (N.I.P.-Y.)

**Keywords:** coumarins, 2-oxo-2*H*-1-benzopyrans, 2-oxo-2*H*-chromenes, 1,2-bezoxaphosphorines, 1,2-bezoxaphosphorines-3-phosphonic acid, 1,2-phosphorinines, conjugate addition, [2+2] cycloaddition, [3+2] cycloaddition

## Abstract

Coumarins are an important class of natural heterocyclic compounds that have attracted considerable synthetic and pharmacological interest due to their various biological activities. This review emphasizes on the synthetic methods for the preparation of dialkyl 2-oxo-2*H*-1-benzo- pyran-3-phosphonates and alkyl 1,2-benzoxaphosphorin-3-carboxylates. Their chemical properties as acceptors in conjugate addition reactions, [2+2] and [3+2] cycloaddition reactions are discussed.

## 1. Introduction

Drug discovery plays an important role in the development of modern society as well as the growth of the pharmaceutical and chemical industry. A key step in this process is the identification of the compound properties and activities while planning its molecular structure.

The pyran group is characteristic of a great diversity of compounds possessing different pharmacological properties. Coumarins, 2-oxo-2*H*-1-benzopyrans or 2-oxo-2*H*-chromenes are an important class of natural heterocyclic compounds. The coumarin moiety could be found in different plants’ secondary metabolites. They play a major role in the proper functioning of the individual plant parts. Moreover, coumarin derivatives have gained considerable synthetic and pharmacological interest due to their various biological activities like antitumor, anti-HIV, antimicrobial, anti-cancer, serine protease inhibition, vasorelaxant and antioxidant activity [1,2,3,4,5].

**Figure 1:**
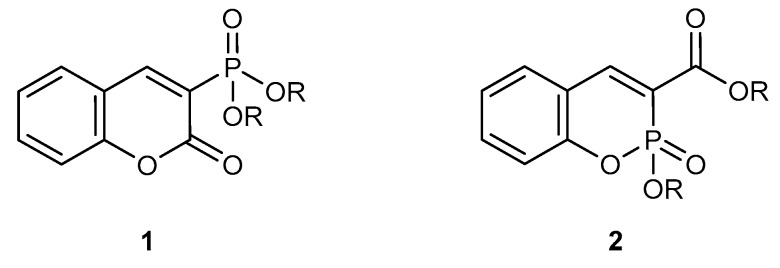
Phosphorus-containing structures **1** and its phosphoroheterocyclic analogue **2**.

**Figure 2:**
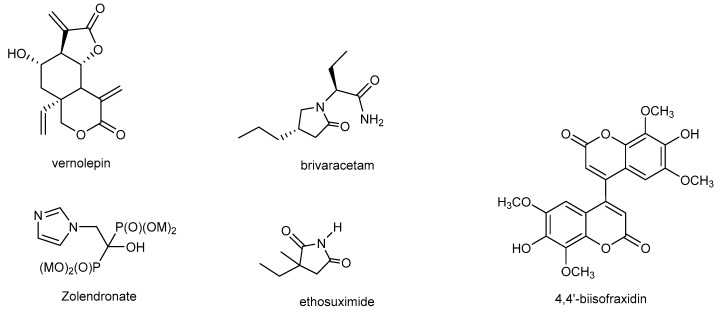
Biologically active compounds and drugs.

Phosphorus-containing structures such as **1** and its analogue **2,** presented in Figure 1, are of a great importance in the areas of pharmacology, chemistry and agriculture due to the similarity of phosphorus compounds to the naturally occurring carboxylic acid derivatives and their possible application in diverse biological systems. Many research papers have illustrated that the chemical behavior of coumarins depends mainly on the substituent at position C-3 in the lactone ring. A phosphoryl group in this position should enhance the biological activities of the resulting 3-phosphonocoumarins as well as influence the chemical properties owing its electron-withdrawing characteristic. Therefore, the combination of the two fragments—a coumarin system containing a phosphoryl group—could open a route to a new class of compounds, which structures might possess wide spectrum of biological activities due to the presence of the different functional groups. In fact, the biological activity of the two represented isomers on Figure 1 is less studied. The great interest to these compounds is due to their application as important precursors for compounds with proven pharmacological properties. Structures of compounds possessing pharmacophore fragments and exhibiting anti-cancer, anti-inflammatory, anti-arthritic and anticonvulsant activity are presented in Figure 2 [6,7,8,9,10,11,12,13].

The Knoevenagel reaction was the first to be applied in the synthesis of phosphonocoumarins as many other 3-substituted coumarins. In the next decades the interest in those derivatives has increased and new synthetic methods have been developed. The first 1,2-bezoxaphosphorines were obtained as side products via lactonization conditions. Therefore, further procedures illustrate alternative routes for their preparation. Surprisingly, only one review paper, published in 2004 [14], appeared presenting some of the properties of these compounds. The aim of this review is to emphasize on the synthetic methods for dialkyl 2-oxo-2*H*-1-benzopyran-3-phosphonates and alkyl 1,2-benzoxaphosphorin-3-carboxylates preparation. The particular interest towards this class of organic compounds is due to their potential application as acceptors in different organic reactions with nucleophillic reagents and 1,3-dipolar cycloaddition reactions as well as to their application as intermediates in the synthesis of products of practical interest proving their biological activity as new therapeutics.

## 2. Synthesis and Some Reactions of Dialkyl 2-oxo-2*H*-1-benzopyran-3-phosphonates 1

### 2.1. Synthesis of Dialkyl 2-oxo-2H-1-benzopyran-3-phosphonates **1**

Since the first synthesis of 3-diethylphosphonocoumarin (**1a**) by Robinson and Addison in 1966 [15], many research groups have been interested in its chemical behavior and various synthetic strategies to access this molecule have been presented. There are several methods described in the literature for the synthesis of diethyl 2-oxo-2*H*-1-benzopyran-3-phosphonates **1** and their derivatives. These protocols can be grouped according to the method or reaction used into Knoevenagel condensations [15,16,17,18,19,20,21], applications of phosphoryl ketenimines [22] or vinylphosphonates [23,24,25,26,27] as precursors, catalytic [28,29,30,31,32] or electrochemical [33,34,35] phosphorylation, protocols using three-component and tandem coupling reactions [36,37,38] or through rearrangement processes [39]. 

#### 2.1.1. Synthetic Protocols Applying Knoevenagel Reaction

**Scheme 1:**
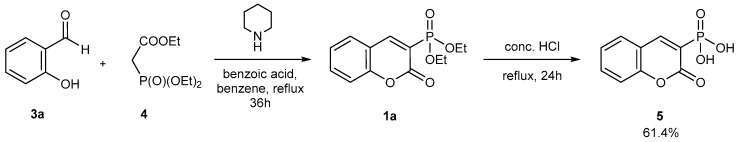
Knoevenagel condensation reaction of salicylaldehyde (**3a**) with triethyl phosphono- acetate (**4**).

3-Diethylphosphonocoumarin (**1a**) was obtained via a Knoevenagel condensation reaction [15] of salicylaldehyde (**3**) with triethyl phosphonoacetate (**4**) under basic conditions. However, the formed product could not be purified due to its high boiling point and the structure of **1a** was determined on the basis of the hydrolyzed product **5** (Scheme 1).

**Scheme 2:**
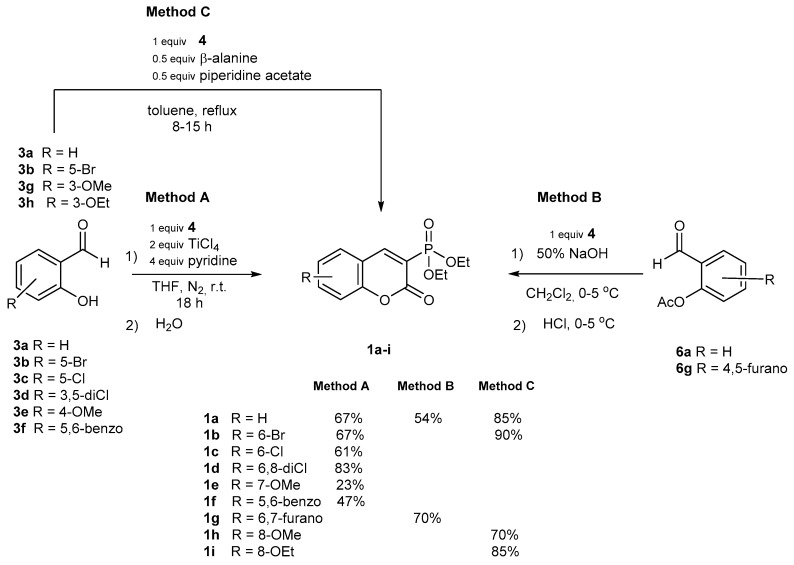
Different synthetic methods for the formation of 3-dialkylphosphonocoumarins **1**.

In 1985 Singh and Rogers [16] reported a modified procedure for the synthesis of substituted 3-diethylphosphonocoumarins by using triethyl phosphonoacetate (**4**) and series of salicylaldehydes **3a**–**f** (Scheme 2, Method A). The reaction was accomplished in 18 h using titanium tetrachloride/pyridine as a catalytic system and tetrahydrofuran as a solvent. The structures of isolated 3-dialkylphosphonocoumarins **1a**–**f** were characterized for the first time by ^31^P-NMR spectra and for **1b** by ^1^H-NMR.

Bouyssou and Chenault studied the formation of coumarins **1a** and **1g** under liquid/liquid phase transfer conditions at low temperatures [18]. The condensation reaction between acetylated hydroxyaromatic aldehydes **6a** and **6g** with triethyl phosphonoacetate (**4**) was carried out applying sodium hydroxide as a base (Scheme 2, Method B). The Knoevenagel condensation products **1a** and **1g** were isolated in good yields (Scheme 2, Method B). 

Falsone et al. [19] applied piperidine acetate/β-alanine as catalyst for the initiation of the lactonization process. The reaction between substituted salicylaldehydes **3a**–**b**, **3g**–**h** and triethyl phosphonoacetate (**4**, Scheme 2, Method C) resulted in the formation of 3-diethylphosphono- coumarins **1a**,**b** and **1h**,**i** in high yields. The applied catalyst demonstrated excellent activity in the Knoevenagel condensation in comparison with the other catalytic systems used in Method A and Method B.

**Scheme 3:**
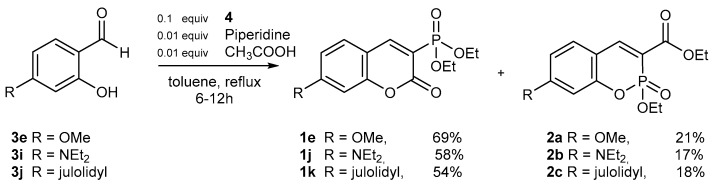
Knoevenagel condensation reaction with piperidine as catalyst.

Chen and coauthors [17] were the first to report the formation of alkyl 1,2-benzoxa- phosphorin-3-carboxylates **2** via Knoevenagel condensation. The reaction of triethyl phosphonoacetate (**4**) and electron-donating group-substituted salicylaldehydes **3e** and **3i**,**j** was catalyzed by freshly prepared piperidinium acetate. Thus, not only the corresponding 3-diethylphosphonocoumarins **1e** and **1h**,**i** were isolated and characterized, but also their analogues **2a**–**c** in which a P-atom replaced the α-pyronyl carbon atom (Scheme 3). In Section 3. of the article the specific methods for the synthesis of 1,2-benzoxaphosphorines and their derivatives are discussed.

**Scheme 4:**
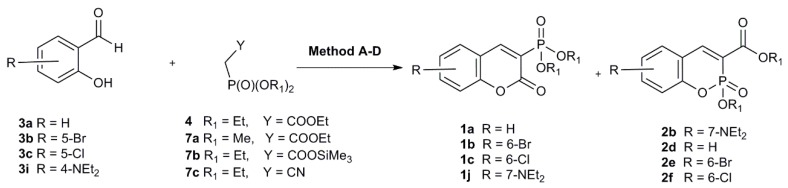
Knoevenagel condensation reaction with organic and inorganic catalysts.

Few years later another approach presented the synthesis of 3-diethylphosphonocoumarines and 1,2-benzoxaphosphorines applying various CH-acidic components. Bojilova et al. [20] performed the Knoevenagel reaction under modified conditions using both organic and inorganic catalysts (Table 1, Scheme 4). The main contribution of the study was the analysis of the ratio between the two chemoisomers and the possible mechanism of the reaction. 

3-Diethylphosphonocoumarines **1a**–**c**,**j** and 1,2-benzoxaphosphorines **2b**–**f** were prepared in solution (toluene and ethanol) and in the presence of an organic base (Method A, A^1^, C, D; Table 1). Different adsorbents (Al_2_O_3,_ zeolites, molecular sieves and Florisil) or titanium tetrachloride (Method B; Table 1) were also applied as catalysts and the only isolated product was the corresponding substituted 3-diethylphosphonocoumarin. 

The detailed results and information are presented in Table 1 where the different reaction conditions, the comparisons of the ratios between the two products and the influence of the substituent R are illustrated. The optimized conditions revealed that the crucial step for the condensation is the azeotropic removal of water. For example the overall yield of 98%, and 84% for **1a**, was achieved when toluene/piperidine and a Dean-Stark short path trap were used for the reaction between triethyl phosphonoacetate and salicylaldehyde. The effective condensation reaction depends on the used CH-acidic component and the electronic effect of the substituent in the aromatic aldehydes. 

**Scheme 5:**
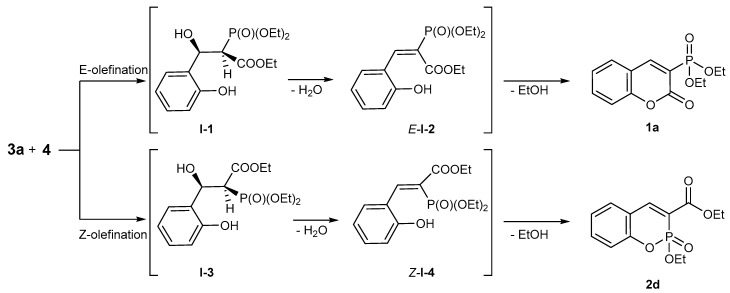
Different reaction paths explaining the ratio of the products **1** and **2**.

The observed regioselectivity is explained on the base of the configuration of the two possible intermediates **I-1** and **I-3** (Scheme 5) which are formed in the addition step. Subsequent *trans*-elimination of a water molecule leads to formation of intermediates *E*-**I-2** and *Z*-**I-4**. The following intermolecular pre-esterification of the phosphoryl or ester group resulted in ring closure to the corresponding 3-diethylphosphonocoumarines or 1,2-benzoxaphosphorines. The preferred *E*-configuration of the intermediate *E*-**I-2** was due to the strong steric interaction between the bulky phosphoryl group and the hydroxyphenyl substituent that could be observed in the intermediate **I-3**. The predominant isolation of 3-diethylphosphonocoumarines over 1,2-benzoxaphosphorines is a result of the formation of the stable *E*-isomer in that reaction conditions. 

**Figure 3:**
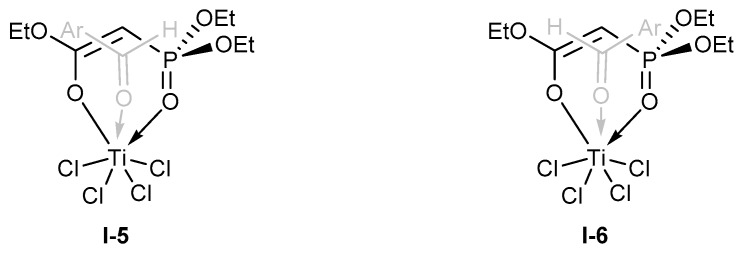
Titanium chelate-complex formation.

The complete regioselectivity of the reaction in the presence of titanium tetrachloride (Method B) or adsorbents was explained by a chelate-complex formation, Figure 3, involving **4** and the titanium salt. An analogous orientation of the reactants on the surface of the adsorbent might also be assembled in that case. Due to the strong steric effect between the phosphoryl and the aromatic group, the formation of intermediate **I-5** was favored and since this steric interaction could appear in the first step of the process, it could prevent the formation of **I-6**. Therefore, the reaction took place as a preferential stereoselective *E*-olefination.

Regulating plant growth activity of a series of 3-diethylphosphonocoumarins and 1,2-benzoxaphosphorines, synthesized in this study, was tested [40] as auxins of different plant seeds. 

**Scheme 6:**
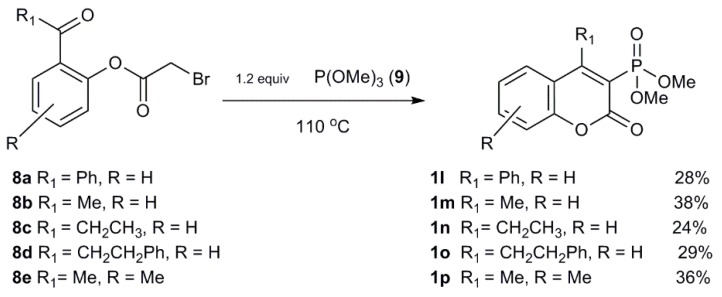
Reaction of 2′-bromoacetoxyphenones **8a**–**e** with trimethyl phosphite (**9**).

During the investigations of the synthesis of new therapeutics, Budzisz et al. performed a reaction of 2′-bromoacetoxyphenones **8a**–**e** with trimethyl phosphite (**9**, Scheme 6) and coumarin compounds were isolated as minor products [21]. The authors elaborated on the formation of Arbuzov type CH-acidic compounds that proceeded in an intramolecular Knoevenagel condensation resulting in the formation of **1l**–**p**. 

Alkylating effect and cytotoxicity of several phosphorylated coumarins have been studied [41] on some human leukemia cell lines HL-60 and NALM-6. Substituents in third or fourth position in the lactone ring were essential for the increased cytotoxicity.

#### 2.1.2. Synthetic Procedure Including Phosphoryl Ketenimines

**Scheme 7:**
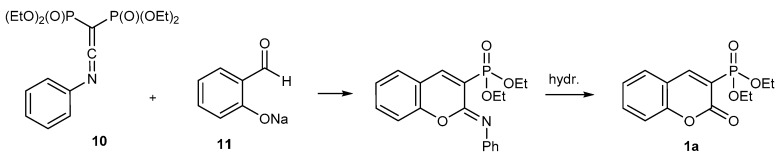
Reaction with compound **10**.

In 1991, Bestmann and Lehnen [22] presented a synthesis of 3-diethylphosphonocoumarin (**1a**) from compound **10** (Scheme 7). Instead of an unsymmetrical CH-acidic component, they used a multistep procedure including methylendiphosphonate to prepare *N*-phenyl-bis(diethyl- phosphono)ketenimine (**10**). In the next synthetic step, sodium 2-formylphenolate (**11**) was used as a Michael donor to form the target molecule. 

#### 2.1.3. Synthetic Procedure Including Vinylphosphonates—Friedel-Crafts Alkylation of Phenols

**Scheme 8:**
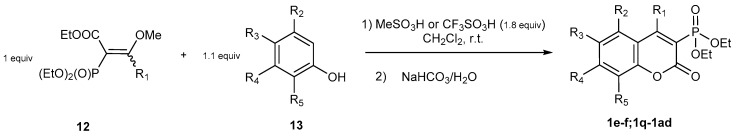
Synthetic method uses vinylphosphonates and phenols.

Another approach for the synthesis of the target 3-diethylphosphonocoumarins **1** was applied by Janecki and coworkers [23,24,25,26,27], using strong acids as catalysts for Friedel-Crafts alkylation of differently substituted phenols followed by spontaneous lactonization (Scheme 8).

From the reactions of 2-diethoxyphosphorylacrylates **12** with substituted phenols or naphthols **13**, series of substituted dialkyl 2-oxo-2*H*-1-benzopyran-3-phosphonates **1e**,**f**, **1q**–**ad** were isolated and characterized. The electrophilic addition was catalyzed by methanesulfonic acid or trifluoromethanesulphonic acid at room temperature. However, strong acidic promoter as trifluoroacetic acid was less effective in the described conditions. This synthetic procedure presents an alternative method in which in higher yields the target compounds **1e**,**f**, **1q**–**ad** were obtained but the conversion time for the reaction was long especially in the presence of methanesulfonic acid (Table 2). The applied conditions were not suitable for the formation of **1t** and **1z** where the starting compound was α-naphthol. 

In all performed reactions, a full regioselectivity to C-addition products was observed, which further undergo intermolecular cyclization resulting in the formation of substituted 3-diethylphosphonocoumarins in moderate to very good yields. 

#### 2.1.4. Phosphorylation of Coumarins

##### Catalytic Phosphorylation

**Scheme 9:**
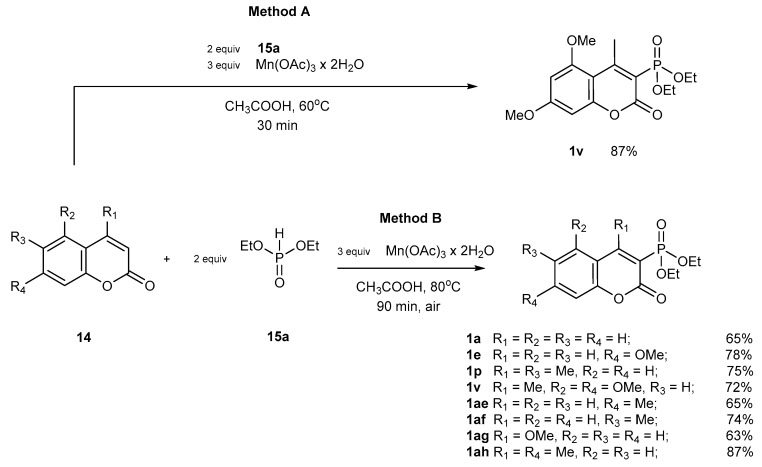
Catalytic phosphorylation with Mn(OAc)_3_.

The direct C-H functionalization appeared to be an atom-economical and environmentally friendly synthetic method. Lately manganese(III) acetate has been introduced as a new reagent for initiation of phosphorus radicals. Zou and coauthors [28] presented a new approach for phosphonocoumarins using direct phosphonation of C-H bond to sp^2^-hybridized carbon (Scheme 9). The reaction of coumarin **14** with diethylphosphite (**15a**) in the presence of Mn(OAc)_3_ for 30 min gave 3-diethylphosphonocoumarin **1v** as a product of regioselective α-phosphonation in yield of 87% (Scheme 9, Method A). The selectivity of the reaction to α-phosphorylation product was not only a result of dialkylphosphonyl radicals formation but also due to the differentiation of α-position in arylalkene as a high electron density center and stabilization of the benzyl radical generated after the attack at the α-position.

The same group of authors [29] reported a modified procedure for the synthesis of a series of substituted coumarin systems. The presented method displayed a regioselectivity for the C-3 position in the coumarin and formation of compounds **1a**, **1e**, **1p**, **1v**, **1ae**–**ah** in yields of 63 to 87%, (Scheme 9, Method B). Substituents on the phenyl ring or at position C-4 at the benzopyran had no significant impact on the yields of the target molecules.

**Scheme 10:**
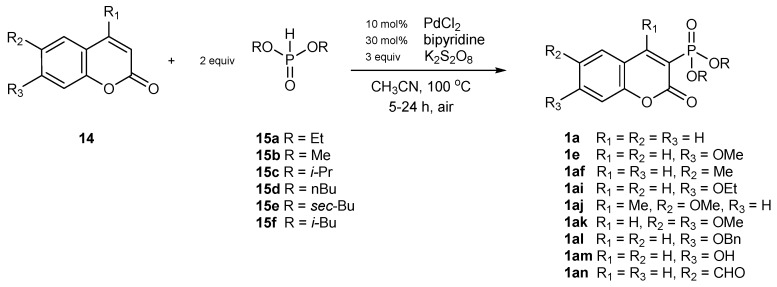
Catalytic phosphorylation with Pd-complexes.

One year later, Wu and coworkers [30] reported a new protocol for selective synthesis of substituted dialkyl 2-oxo-*2H*-1-benzopyran-3-phosphonates **1a**, **1e**, **1af**, **1ai**–**an** via a direct Pd-catalyzed phosphonation of coumarins (Scheme 10). 

The reactions were performed by using 2,2′-bipyridine as a ligand, PdCl_2_ as a transition-metal source, potassium peroxodisulfate as an oxidant and acetonitrile as a solvent media. Different dialkyl *H*-phosphonates **15a**–**f** were tested in the optimized reaction conditions and the di-*iso*-propyl (yield 56%) and di-*sec*-butyl (yield 59%) *H*-phosphonates displayed better C-H phosphonation activity toward the unsubstituted coumarin in comparison with the other dialkyl phosphonates (R = Me 44%; R = Et 48%; R = *n*-Bu 47%; R = *i*-Bu 51%). The coumarins bearing electron-donating groups reacted faster and gave better yields than their analogues possessing electron-withdrawing groups. Substituents as Me and OMe at position C-6 in the benzene ring showed significant influence on the phosphorylation reaction yielding **1** in 52% and 63%. The coumarins **1aj** and **1k** were prepared in 10% lower yields than **1e**.

**Scheme 11:**
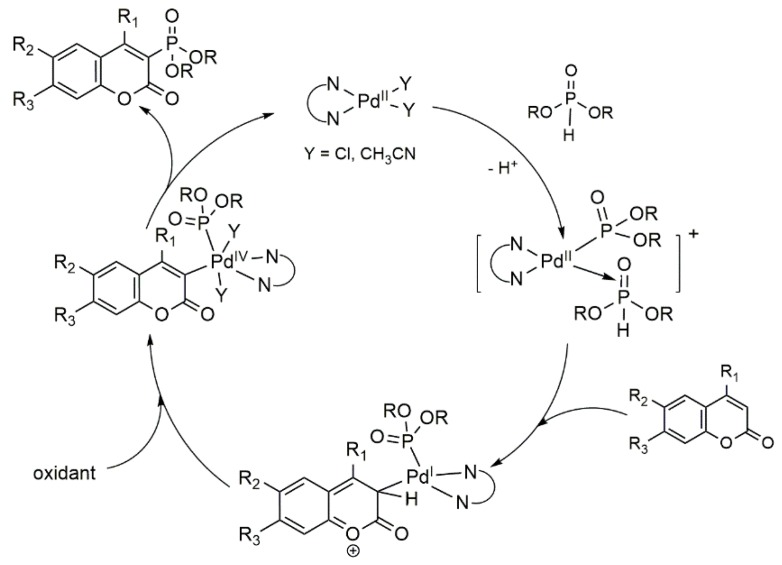
Proposed mechanism for the synthesis of substituted dialkyl 2-oxo-2*H*-1-benzopyran-3-phosphonates.

It is worth noting that the reaction conditions were tolerant of systems with hydroxyl and formyl groups, even though the yields were low, 34% and 27%, respectively. Use of a radical scavenger (2,2,6,6-tetramethylpiperidin-1-yl)oxidanyl (TEMPO) provided evidence for a possible mechanism of the reaction. In the performed parallel reactions the formation of the 3-diethylphosphonocoumarin allowed the possibility of a radical mechanism to be discarded (Scheme 11).

**Scheme 12:**
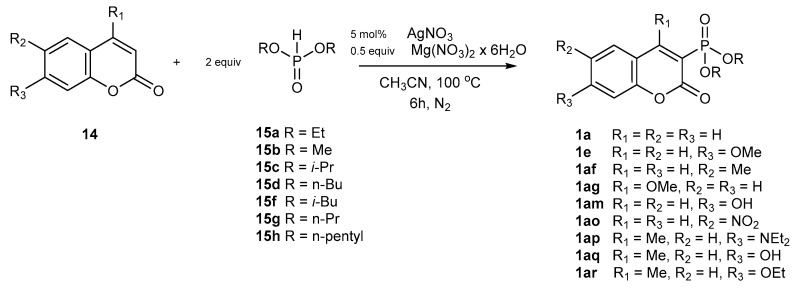
Ag-catalyzed phosphorylation reaction.

An efficient regioselective silver-catalyzed direct Csp^2^-H radical phosphorylation of coumarins was presented by Mao et al. [31]. Phosphorus-containing compounds **1a**, **1e**, **1af**–**ag**, **1am**–**ar** were prepared in a reaction of coumarins **14** with different dialkyl *H*-phosphonates **15a**–**d**, **15f**–**h** under mild reaction conditions in the presence of catalyst AgNO_3_/Mg(NO_3_)_2_ × 6H_2_O (Scheme 12). The metal-promoted synthetic protocol derived the expected products **1** with moderate to good yields (45–65%). 

**Scheme 13:**
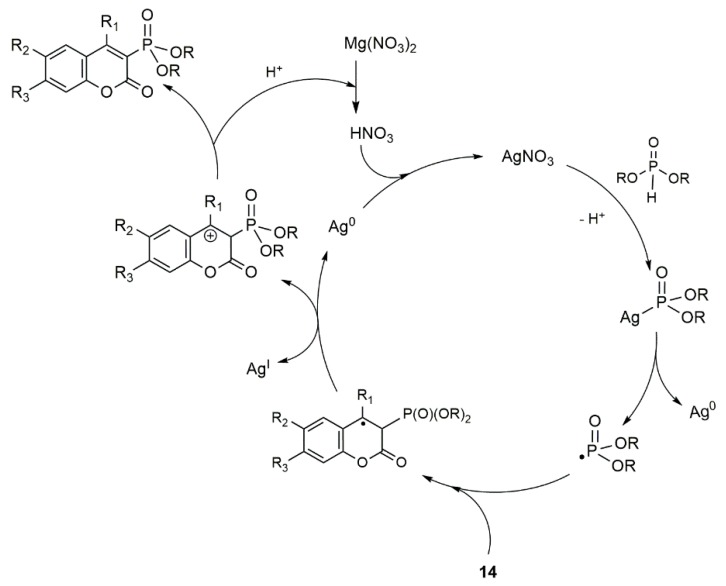
The proposed radical mechanism of the Ag-catalyzed phosphorylation reaction.

According to the proposed mechanism (Scheme 13) Ag(I) promotes the initiation of phosphoryl radicals which implied that the C-H phosphorylation of coumarins—experience a radical reaction path. Indeed, formation of 3-dialkylphosphonocoumarin was not observed in the presence of radical scavenger TEMPO that proved the radical mechanism of the process.

In the conditions of radical phosphonation coumarins with electron-donating groups proceeded with better results than their analogs having electron-withdrawing substituents. The diethylamino group was found to be a substituent that activated the coumarin system toward the C-P formation, thus, highest yields were observed in this case—65%. The electron-withdrawing effect of the nitro group slightly deactivated the benzopyran system and therefore product **1ao** was obtained in yield of 45%. Interestingly, coumarin **1am** was afforded in nearly doubled yield comparing with the previous procedure. The di-*iso*-propyl (60%) and dimethyl (62%) *H*-phosphonates displayed better activity for the coumarin **1a** in comparison with the other dialkyl phosphonates (R = Et 55%; R = *n*-Bu 49%; R = *i*-Bu 54%; R = *n*-Pr 50%; R = *n*-pentyl 45%).

**Scheme 14:**
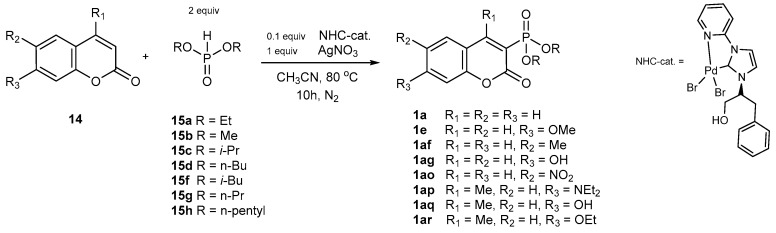
Phosphorylation reactions by N-heterocyclic carbene palladium complexes.

A year later, Mao et al. reported [32] a modified selective protocol for the phosphorylation of coumarins using N-heterocyclic carbene palladium complexes. The catalytic activity of different NHC-complexes was implied in the reaction. The complex presented in Scheme 14 showed the best catalytic efficiency in the phosphorylation reaction. A wide range of dialkyl *H*-phosphonates **15a**–**d**, **15f**–**h** reacted smoothly with coumarins **14** resulting in the complete regioselective synthesis of the target molecules **1a**, **1e**, **1af**, **1am**–**ar** in moderate to high yields.

The optimized conditions for the selective phosphorylation of coumarins **14** included a combination of catalysts—Pd-bearing component and Ag(I) salts—thus, the highest yields were reported. Substituents at C-7 or C-4 in the benzopyran system favored the phosphorylation reaction (86–89%), though substituents at position C-6, either electron-withdrawing (41%) or electron-donating groups (36%), resulted in lower yields. The results for the coumarin **1a** remained higher using di-*iso*-propyl (70%) and dimethyl (68%) *H*-phosphonates than other dialkyl phosphonates (R = Et 64%; R = *n*-Bu 44%; R = *i*-Bu 46%; R = *n*-Pr 46%; R = *n*-pentyl 52%).

##### Electrochemical Phosphorylation

**Scheme 15:**
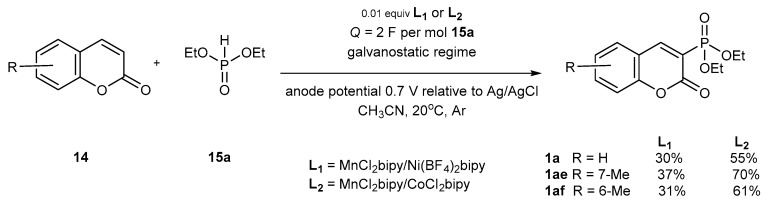
Electrochemical phosphorylation of coumarins with diethyl *H*-phosphonate.

The preparation of 3-dialkylphosphonocoumarins through a direct phosphorylation of C-H bond of the aromatic system remains one of the used synthetic approaches that corresponds to the principals of green chemistry–atom economy, single step processes, low amounts of waste products, etc. Recent approaches for the synthesis of 3-dialkylphosphonocoumarins include electrochemical initiation of the phosphorylation. A new method for C-P bond formation at position C-3 in the benzopyran system was reported in 2016 by Khrizanforov et al. [33,34]. The synthetic protocol was based on an oxidation process of aromatic compounds **14** and diethyl phosphonate (**15a**) in the presence of bimetallic catalytic systems (Scheme 15). Two bimetallic systems as the pairs MnCl_2_bipy/Ni(BF_4_)_2_bipy and MnCl_2_bipy/CoCl_2_bipy were used where 2,2′-bipyridine was an additional ligand. The reaction took place at room temperature by applying equimolar ratio between the coumarin and the used phosphonate **15a**. Coumarins, bearing electron-donating groups, gave better yields in phosphonation reactions in comparison with the unsubstituted benzopyran system (Scheme 15).

**Scheme 16:**
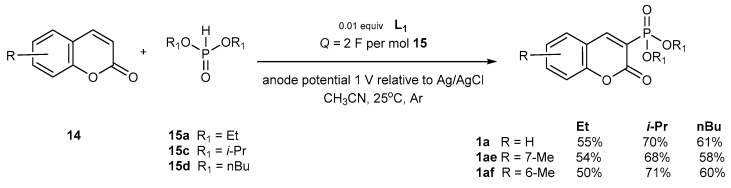
Electrochemical phosphorylation of coumarins with dialkyl *H*-phosphonate.

Subsequent investigations by Khrizanforov et al. [35] for the preparation of coumarin phosphonates in the presence of MnCl_2_bipy/Ni(BF_4_)_2_bipy showed changes in the electrochemical parameters by applying three dialkyl *H*-phosphonates (Scheme 16). In this study di-*iso*-propyl phosphonate resulted in yields of up to 70%. An interesting circumstance is that the yields were obtained after one-day reaction mixture storage before electrolysis, which was necessary for the formation of the metal phosphonate complex. Full conversion of the dialkyl *H*-phosphonate was observed under the described conditions.

#### 2.1.5. Synthetic Protocols Involving Coupling Reactions

**Scheme 17:**
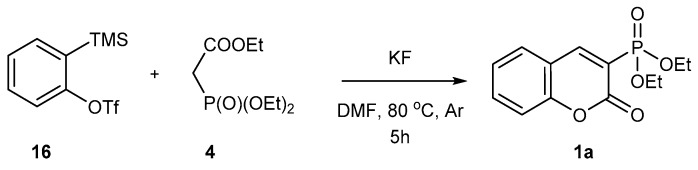
Synthesis of 3-diethylphosphonocoumarin under coupling reaction.

Different approach for the synthesis of the target 3-dialkylphosphonocoumarins includes three-component coupling of arynes, dimethyl formamide (DMF) and CH-acidic component **4** (Scheme 17). 

**Scheme 18:**
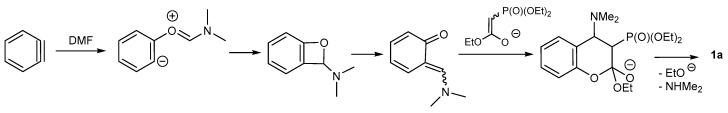
Possible mechanism of the reaction involving *ortho*-quinone methide.

The reported protocol [36] represents a reaction between an in situ generated benzyne fragment (Scheme 18) and an active methylene compound bearing a diethylphosphoryl group in DMF at 80 °C. The compound **1a** was produced in 74% yield (Scheme 17). 

The course of the reaction was switched when the solvent was changed. The nucleophilic attack of the carbonyl oxygen of DMF molecule to benzyne was a probable initiator of the reaction followed by the formation of *ortho*-quinone methide intermediate (Scheme 18).

**Scheme 19:**
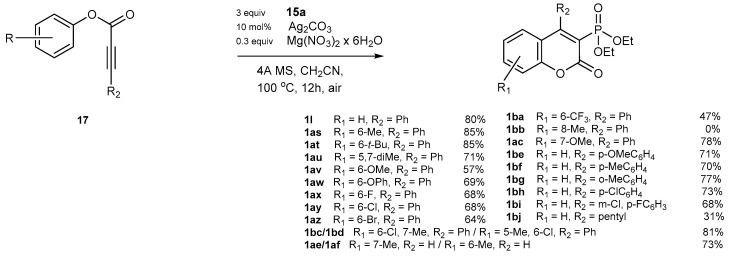
Transition-metal-catalyzed cross-coupling synthetic protocol for the formation of phosphorus-containing coumarin systems.

Currently several utilized methods of C-P bond construction as an electrophilic phosphorus reagent and transition-metal-catalyzed cross-coupling synthetic protocols for the formation of phosphorus-containing coumarin systems are listed. Another approach relied on a domino reaction via an intermolecular addition of P-centered radicals to alkynes followed by fast cyclization onto the aromatic π-system [37]. The tandem radical phosphorylation-cyclization reaction of readily prepared phenyl alkynoates **17** with diethyl *H*-phosphonate substrate **15a** was investigated in the presence of combination of Ag_2_CO_3_, Mg(NO_3_)_2_x6H_2_O in CH_3_CN for 12 h to afford the corresponding 3-dialkylphosphonocoumarins **1l**, **1as**–**bj** (Scheme 19).

The steric effect had strong impact on the accomplishing of the radical tandem reaction. For example, the formation of compound **1bb** was not observed due to the hindrance of the methyl group on the *ortho*-position of the phenoxy ring in **17**. By applying aryl alkynoates bearing 4-Me, 4-OMe or 4-Cl substituents at the *para*-position in the phenoxy ring the authors studied the regioselectivity of the reaction. Products **1bc**/**1bd** and **1ae**/**1af** were produced in a mixture of two isomers. Product **1ac** was formed in high yield (78%) with complete regioselectivity of the process. The products formation was as a result of cyclization reaction preferably occurring at the opposite site of the substituent.

**Scheme 20:**
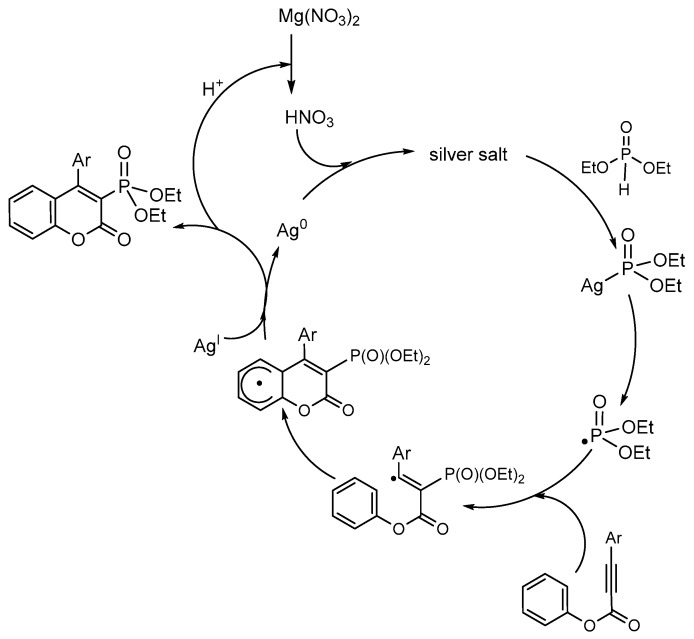
The suggested mechanism of the observed cross-coupling transformation.

The cascade reaction was accomplished smoothly with dimethyl *H*-phosphonate and di-*iso*-butyl *H*-phosphonate yielding the corresponding product **1l** in 70% and 90%, respectively. A series of controlled experiments using TEMPO as a radical scavenger and experiments with deuterium labelling were executed to highlight the mechanism of the observed transformation (Scheme 20). The results demonstrated that the cleavage of the C-H bond on the phenoxy ring was not involved in the rate-determining step.

**Scheme 21:**
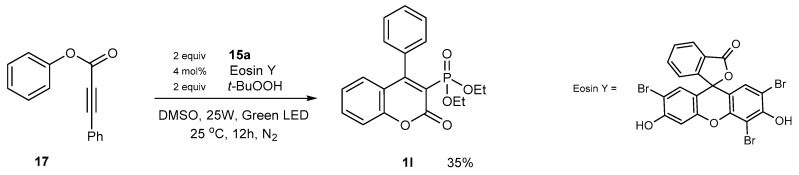
Cascade-radical approach for the formation of 3-functionalized coumarin.

A photochemical technique for the synthesis of derivative **1l** uses a cascade radical addition of phosphorus nucleophiles to aryl propiolates. Xu et al. [38] employed catalytic quantities of the commercially available Eosin Y (EY) as a photocatalyst and *tert*-butyl-hydroperoxide as an oxidant (Scheme 21). 

#### 2.1.6. Rearrangement Reactions

**Scheme 22:**
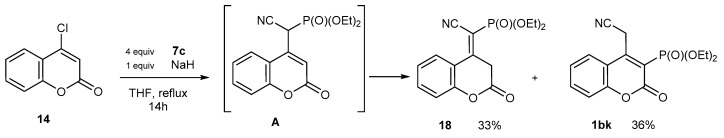
Rearrangement reaction of intermediate **A**.

CH-acidic component as diethyl cyanomethylphosphonate (**7c**) was used in a reaction with some α- and β-monohalocarbonyl compounds [39]. As a result of nucleophilic aromatic substitution an intermediate **A** was formed from 4-chlorocoumarin (**14**) with **7c** in the presence of an equimolar amount of NaH in THF, Scheme 22. Tautomerization to vinylphosphonate **18** or thermal rearrangement to the 3-diethylphosphonocoumarin **1bk** were next steps in the intermediate **A** transformation. 

The observed rearrangement is expected because the chemical properties of 3-substituted coumarins frequently are pursued by such benzopyran-2-oxochroman transformation [42,43,44,45,46].

### 2.2. Reaction of Dialkyl 2-oxo-2H-1-benzopyran-3-phosphonates **1**

**Figure 4:**
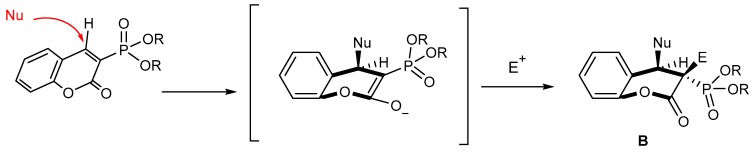
Nucleophilic addition reactions to 3-dialkylphosphonocoumarin.

The presence of a conjugate π-system in the lactone ring of 2-oxo-2*H*-1-benzopyran derivatives **1** implies their involvement in nucleophilic addition reactions (Figure 4). Various factors as substituents in the lactone ring, the strength of the used nucleophile, the solvent, etc. influence the reaction rate, the type and the yields of the obtained products. There are many examples in the literature presenting a coumarin structure as an excellent Michael acceptor [42,47,48,49] producing adducts of type **B** as a main product (Figure 4).

#### 2.2.1. Reactions with Nucleophilic Reagents

**Figure 5:**
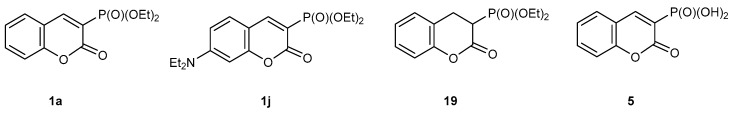
Structures of phosphorus-containing coumarins that were object of theoretical investigations.

Chemical properties of diethyl 2-oxo-*2H*-1-benzopyran-3-phosphonate (**1a**), diethyl 3,4-dihydro-2-oxo-*2H*-1-benzopyran-3-phosphonate (**19**), 2-oxo-*2H*-1-benzopyran-3-phosphonic acid (**5**) and diethyl 7-N,N-diethylamino-2-oxo-*2H*-1-benzopyran-3-phosphonate (**1j**, Figure 5) were the object of experimental and theoretical reactivity parameter studies distinguishing the electrophilicity of the center in the coumarin system by atomic electrostatic potential and XPS binding energies while electronic localization was examined on the base of atomic Fukui indices [50]. The calculated electronic structures have characterized with large negative charge on C-3-atom whereas C-4 atomic charge has values close to zero. Obtained 2p-binding energies for P-atoms described identical local environment for the coumarins. On contrary, in 7-diethylamino substituted compound energy was low probably due to interaction between N- and P-substituents displayed as higher electronegativity at P-atom. Calculated atomic electrostatic potentials also showed that the electrophilicity of the reaction centers in the coumarin system increases in the presence of phosphonic group, especially in coumarin-3-phosphonic acid **5**. Electron donating group at C-7 increased electrophilicity of the C-7 carbon but at the same time C-3 and O-2 atoms from the lactone ring increased their nucleophilicity.

As a result the the phosphorus-containing coumarins C-3 atoms in these chemical structures were defined as soft centers. Indeed, Fukui indices showed increased values for C-3, especially when a phosphoryl and diethylphosphoryl group is bound, and characterized the center as hard. Actually, phosphorus-containing substituents did not influence the electron localization at the C-3 atom like withdrawing groups but were most like a hydrogen atom due to the similar electronegativity of H- and P-atoms. Indices for the carbon atoms characterized C-3 atoms as hard centers compared with C-2. Furthermore, the studied parameters anticipated the reactivity in the lactone ring accounting for the lower nucleophilicity of the O-2 atom compared to C-3, and moreover, the participation in 1,2-addition reactions with nucleophiles.

#### 2.2.2. Reactions with Hydrides

**Scheme 23:**
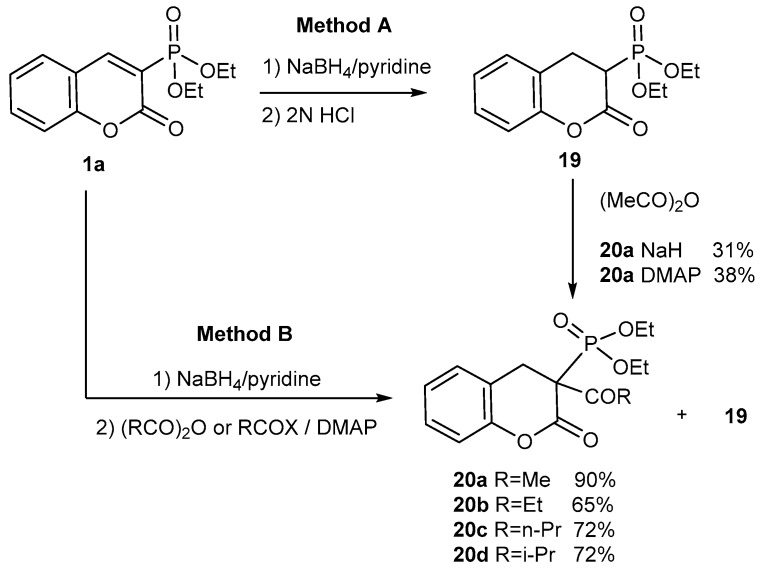
One-pot tandem hydrogenation/acylation reactions with 3-diethylphosphonocoumarin **1a**.

The chemical behavior of diethyl 2-oxo-*2H*-1-benzopyran-3-phosphonate (**1a**) in hydrogenation/acylation reactions under different conditions was studied [51] and high regioselectivity for the C-acylation product **20** was observed. Two reaction paths presented in Scheme 23 demonstrate the advantage of one-pot reactions versus the multistep routes. The first path shown as Method A involved deprotonation of 3,4-dihydroadduct **19** by NaH or DMAP and formation of enolate ion which reacted with anhydride to form the acylated product **20a**. However, the yields were rather unsatisfactory, 31 or 38%. The second approach, Method B, presented a one-pot reaction with in situ generated enolate ion followed by its trapping with a series of anhydrides produced the expected products **20a**–**d** in higher yields (Scheme 23).

The yields of products **20a**–**d** depended strongly on the solvent—pyridine—and the catalyst—DMPA—used in the process. During the optimization of the reaction conditions, the ratio between the anhydrides and DMAP varied from 3:1 to 3:2.2. Acetic, propionic and butyric anhydrides resulted in C-acylated products in 90%, 65% and 72% yield after 2.5 h at −4 °C. For bulky acylating reagents such as *iso*-propyl anhydride, running the reaction at room temperature enhanced the yield of **20d** from 47 to 72%. The steric hindrance between the newly incorporated acyl group and the phosphoryl group at position C-3 determined the outcome of the reaction. 

**Figure 6:**
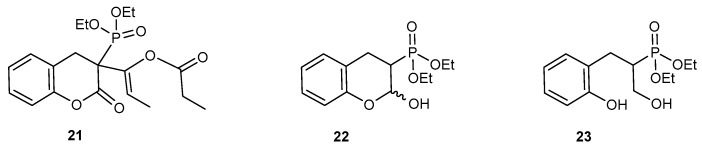
Products isolated from acylation reaction.

Products of subsequent acylation **21** or further hydrogenation **22** and **23** were also isolated, (Figure 6). The predominant presence of dihydro adduct **19** when the reaction was carried out in THF confirmed the importance of the used solvent, and further hydrogenation of **19** resulted in formation of derivatives **22** and **23**. 

#### 2.2.3. Reactions with Organometallic Reagents

**Scheme 24:**
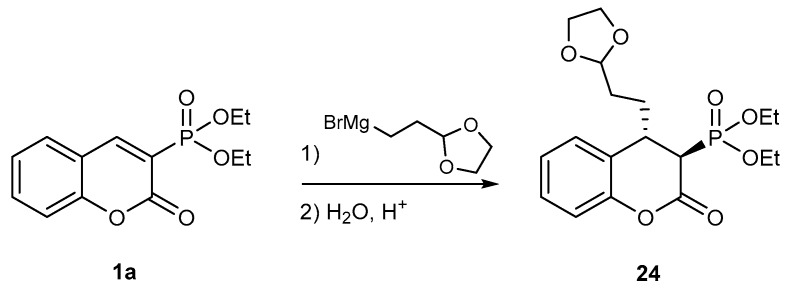
Reaction of 3-diethylphosphonocoumarin **1a** with Grignard reagent.

The first reaction of 3-diethylphosphonocoumarin **1a** with a Grignard reagent was reported by Bestmann and Lehnen [22]. Product **24** was isolated as a result of Michael-type addition reaction (Scheme 24). Though, the reaction took place as stereospecific *cis*-addition to the C_3_=C_4_ double bond and the product was characterized by X-ray spectroscopy, no yields from the performed reactions were given in the article. 

A productive study on the option of applying 3-dialkylphosphonocoumarins **1q**–**t** as key intermediates for the formation of α-methylene δ-lactones was reported by Janecki and co-workers [23,24,25,52]. Compounds like α-alkylidene γ-lactones, α-alkylidene δ-lactones and their analogs with lactam ring display pharmacological properties and in particular strong anti-cancer activity. The majority of the synthesized δ-lactones were tested on human leukemia cell lines NALM-6 and HL-60 as well as MCF-7 breast cancer and HT-29 colon cancer cells.

**Scheme 25:**
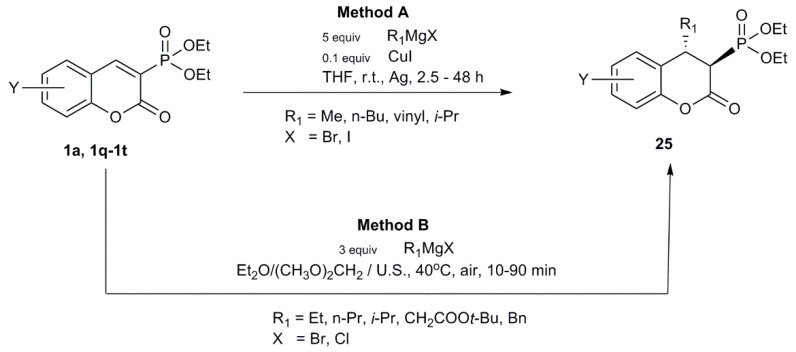
1,4-conjugate addition reactions with organometallic compounds.

In order to obtain the phosphorus-containing adducts for the Horner–Wadsworth–Emmons olefination α-(diethoxyphosphoryl)-δ-lactones **25** were prepared by a 1,4-conjugate addition of organometallic compounds to substituted 3-diethylphosphonocoumarins **1q**–**t** in the presence of catalytic amount of CuI (Scheme 25). The reactions proceeded in a fully diastereoselective manner while the time for conversion depended strongly on the substituents on the benzene ring. For example, when the interaction was performed with **1h** (Y = 8-OMe) using MeMgI as a nucleophile the reaction time was 2.5 h and the yield of **25** was 73%. However, when the same conditions were applied for coumarin **1e** (Y = 7-OMe) the time for its conversion was 48 h and the addition product was obtained in a yield of 69%. In general, using *iso*-propyl and *n*-butylmagnesium halide the yields were in the range of 51 to 93%, whereas for methylmagnesium halide the results were between 68–85%. Interestingly, the structures of isolated compounds were characterized as the *trans*-β-substituted isomers.

Fundamental research on the synthesis of compounds **25** was presented by Nikolova et al. [53]. In this study, ultrasonic waves were used as a promoter of the reaction—Method B, Scheme 25. The sonication method provides an unusual mechanism to generate high-energy chemistry due to the extraordinary temperatures and pressure generated by the collapse of cavitation bubbles, while it can accelerate organic reactions involving metal-mediated processes because of the metal surface activation and the particle size reduction, producing modified metal surfaces and at the same time speeding up the formation of organometallic reagents. The reaction between 3-diethylphosphonocoumarin **1a** and the organomagnesium compound was completed as Michael-type addition and the formation of *trans*-isomers of products **25** were observed. Methods for initiating the reaction—thermal or ultrasonic, were further investigated and a relationship between the yields and the reaction time as well as the applied technique was indicated in Table 3. 

The main disadvantage of the thermal initiation was the lack of reproducibility of the obtained results. By applying the sonication method this obstacle was overcome. The data in Table 3 unambiguously showe the impact of sonication on the decrease of reaction time to 10–40 min, and the increase of the yields of the target molecules. The studied reaction was specified with a complete regioselectivity for the 4-substituted 3,4-dihydrophosphonocoumarins **25**.

A comparison between Method A and Method B displayed distinctly shorter reaction times for the sonicated reactions and absence of requirements as use a specific catalyst, inert atmosphere and high excess of the Grignard reagents used. 

**Scheme 26:**
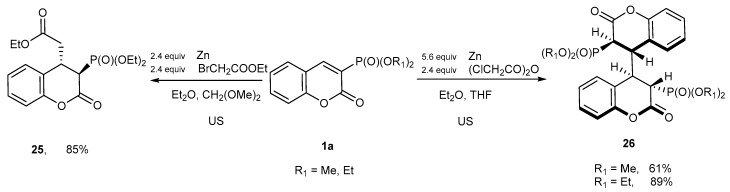
Reactions with organozinc reagents.

Applying Method B to Reformatsky reagents afforded product of type **25 [53]**. However, under ultrasound irradiation, using different organozinc compounds, the dimeric systems **26** were obtained in high yields [54] (Scheme 26).

**Scheme 27:**
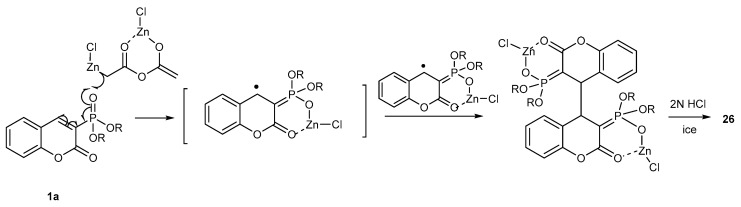
Radical homodimerization mechanism.

Obviously, the processes in Scheme 26 are characterized by two different mechanisms. When compound **25** was formed, the reaction is classified as a Michael-type addition of C-nucleophiles to electron-deficient systems. However, the formation of product **26** cannot be explained by a Michael addition reaction. Therefore, Nikolova et al. investigated the mechanism of the process and it was assumed a radical intermediate formation and a subsequent coupling between the initiated radicals (Scheme 27).

During the investigations on the mechanism of the reaction, the authors tried to synthesize heterodimers by combining coumarin systems with varied reactivity. However, the approaches were not successful and the assumption was that the reaction conditions favored just the homodimerization process whereas the used solvent played a major role in the reaction path. Products **26** were isolated and characterized as *meso*-form structures due to the obtained analytic data from X-ray and NMR spectroscopy.

**Scheme 28:**
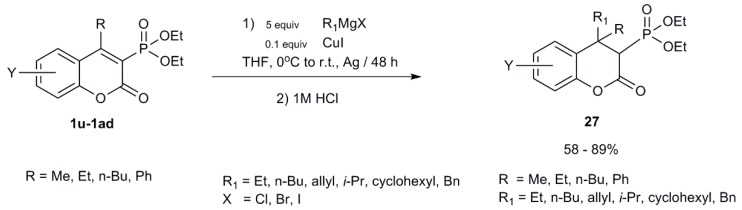
1,4-conjugate addition reactions to 4-substituted 3-diethylphosphonocoumarins.

Applying 4-substituted 3-diethylphosphonocoumarins **1u**–**ad** (Scheme 28), as Michael acceptors another approach toward the investigation of the regioselectivity of the reaction and the possibility for incorporation of a second alkyl substituent in δ-lactones **27** was presented [26,27]. The study was accomplished with a series of organomagnesium reagents (ethyl-, *iso*-propyl-, *n*-butyl-, cyclohexylmagnesium chlorides, allylmagnesium iodide and benzylmagnesium bromide) 4,4-disubstituted 3-diethoxyphosphoryl-3,4-dihydro-2*H*-chroman-2-ones **27** were isolated in moderate to good yields in a mixture of *trans*- and *cis*- isomers. Only in three cases the product was found to be a single *trans*-isomer. 

#### 2.2.4. Three-Component Reactions of Diethyl 2-oxo-2*H*-1-benzopyran-3-phosphonate (**1a**) with Compounds Bearing Carbonyl and Amino Groups

**Scheme 29:**
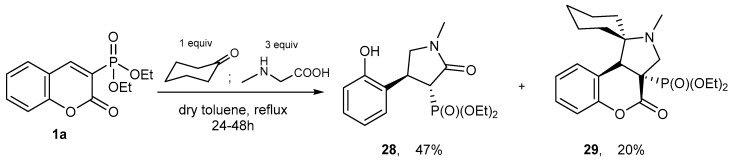
Reaction of **1a** with cyclohexanone and N-methylglycine.

Conjugate addition reactions to 3-diethylphosphonocoumarin **1a** using asymmetric nonstabilized azomethine ylides were investigated by Moshkin et al. [55,56]. The developed approach for the synthesis of products **28** and **29** utilized as starting material the in situ formed ylide derived from cyclohexanone and N-methylglycine (Scheme 29). The structure of compounds **29** resemble the skeleton of adrenoceptor antagonists and are good precursors for new pharmaceutical agents for prostatic hyperplasia. 

The reaction can be illustrated as 1,3-dipolar cycloaddition to the 3-diethylphosphonocoumarin **1a**, therefore product **29** was the expected adduct. Surprisingly, the pyrrolidone **28** was also formed.

**Scheme 30:**
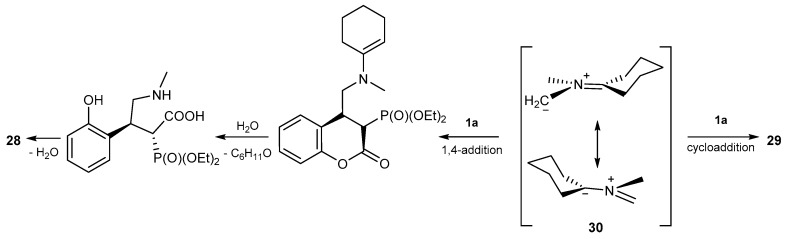
Proposed ylide structure **30** and subsequent interactions.

Due to the steric hindrance, the deactivation of the cationic center and the loss of the dipolar properties of the azomethine structure **30** an initial Michael addition followed by an intramolecular cyclization leading to the formation of the heterocyclic compound **28** was observed (Scheme 30). The participation of cyclohexanone in the formation of pyrrolidone **28** was identified by additional reactions. Without the presence of cyclohexanone no interaction was observed, therefore it played a major role in the formation of **28** as catalyst for the described domino reaction.

**Scheme 31:**
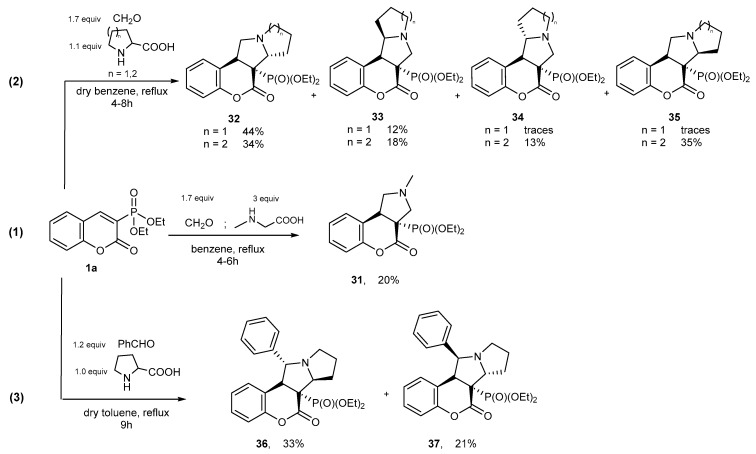
Domino reaction with azomethine ylides.

When the used carbonyl compound was changed to paraformaldehyde (Scheme 31, Reaction (1)), and the reaction was carried out in benzene with azeotropic removal of water, Moshkin and coworkers observed the formation of benzopyranopyrrolidine derivative **31**. The lactone ring conformation in the formed product confirmed the synchronicity of the reaction of nonstabilized azomethine ylides with 3-diethylphosphonocoumarin **1a** resulted in the *cis*-fusion in the new pyrrolidine structure.

Further investigation on the 1,3-dipolar cycloaddition of the ylide, derived from proline and formaldehyde, using diethyl 2-oxo-2*H*-1-benzopyran-3-phosphonate **1a** as a trapping dipolarophile, resulted in the formation of pyrrolizidines **32** as a major product (Scheme 31, Reaction (2), n = 1), a minor diastereomer **33**, and the presence of the other isomers **34** and **35** were determined by ^1^H- and ^31^P-NMR spectroscopy. 

Different selectivity of the cyclization process was observed when pipecolic acid (Scheme 31, Reaction (2), n = 2) was implied in the reaction mixture. The increase of the *endo*-cycloaddition product **35** might be due to the size and the flexibility of the azomethine ylide’s cyclic moiety and the absence of steric hindrance in the transition state.

A large number of pyrrolizidines were observed when benzaldehyde was used as a carbonyl component and only two of the isomers were isolated and characterized as being the major products **36** and **37** (Scheme 31, Reaction (3)).

Phosphorylated azaheterocycles were synthesized [57] by three-component reaction between 3-diethylphosphonocoumarin **1a** with acetone, cyclopentanone or cyclohexanone and benzylamine. These compounds are promising precursors for the preparation of polycyclic δ-lactams possessing potential biological activity.

**Scheme 32:**
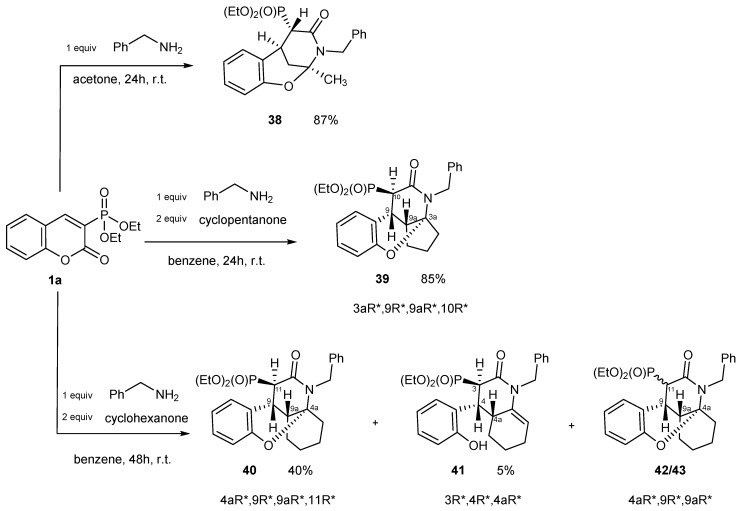
Synthesis of polycyclic δ-lactams.

The reaction of 3-diethylphosphonocoumarin **1a** with benzylamine and acetone, proceeded at room temperature within one day and the desired product **38** was isolated as a single diastereomer in 87% yield (Scheme 32). The structure and absolute configuration of the product was determined by single crystal X-ray crystallography.

Interestingly, only one product was observed when 3-diethylphosphonocoumarin **1a** reacted with cyclopentanone and benzylamine at room temperature at benzene as a solvent. The isolated product **39** was identified as benzoxazocine isomer with H-C(10), H-C(9)- *trans* and H-C(9), H-C(9a)-*cis* relative configuration. Using X-ray analysis a 3aR*,9R*,9aR*,10R* configuration was assigned (Scheme 32).

It should be noted that the reaction of **1a** with benzylamine and cyclohexanone was not stereoselective like the previously mentioned reactions and a mixture of four isomers **40**:**41**:**42**:**43** in a ratio of 1:0.25:0.25:0.1 were isolated. 

The authors used enantiomerically pure (*R*)- and (*S*)-phenylamines as reagents in order to determine the absolute stereochemical course of the reactions. However, even under this approach the unambiguously assignment of the relative configuration of the stereogenic centers C-9 and C-9a in **39**–**43** was not achieved. When adducts were transformed into their corresponding α-methylene-δ-lactams the configuration was distinguished.

##### Reactions with CH-acidic Compounds

**Scheme 33:**
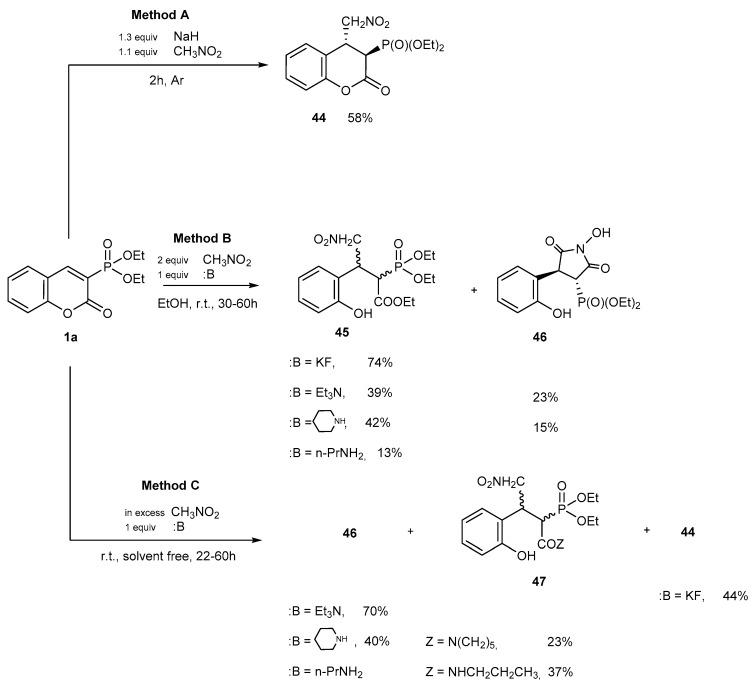
Michael-type addition of nitromethane under different reaction conditions.

CH-acidic compounds were also used as nucleophiles in conjugate addition reactions with 3-diethylphosphonocoumarin **1a** and its derivatives. For example, Janecki et al. [52] applied the sodium salt of nitromethane as an alternative reagent in a Michael type addition reaction to give coumarin **1a**. The reaction proceeded in 58% conversion of the starting material and product **44** was isolated as a single diastereomer with a pseudo-axial disposition of the phosphoryl and nitro groups (Scheme 33, Method A).

**Scheme 34:**
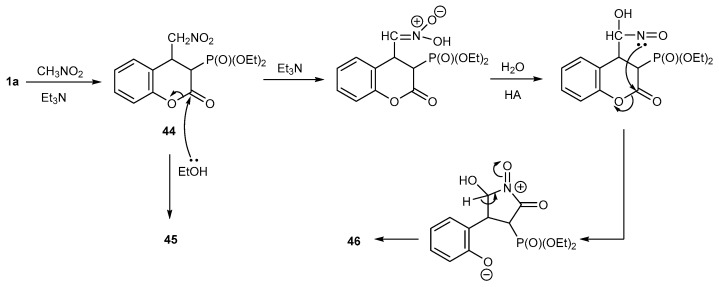
Proposed mechanism for the formation of **45** and **46**.

The interaction between coumarin **1a** and nitromethane using different bases (KF, Et_3_N, C_5_H_11_N) in a protic solvent (EtOH) led to formation of compounds **45** and **46** (Scheme 33, Method B) [58]. Product **45** was isolated in an overall yield of 74% as a mixture of two *trans*-isomers in 1:1.25 ratio when potassium fluoride was used as a base. The transformation involved a 1,4-conjugate addition of nitromethane to the activated double bond in coumarin **1a** and a subsequent attack of a solvent molecule to the carbonyl group of the lactone ring. However, when using piperidine or propylamine an additional product 1-hydroxy-4-(2′-hydroxyphenyl)-2,5-dioxopyrrolidin-3-yl-phosphonate (**46**) was isolated as a single isomer. The mechanism of formation of **46**, shown in Scheme 34, was a result of a base-catalyzed tautomeric transformation of the Michael-type adduct **44** followed by a Nef-reaction rearrangement.

The subsequent synthetic protocol, Method C, Scheme 33, illustrated the conjugate reaction with nitromethane in free solvent conditions. The results emphasized the influence of the bases on the reaction path and on the product ratio. The primary amine led to product **47**, whereas secondary amines promoted formation of **46** and **47**. Compound **46** was isolated as an only product in very good yields when triethylamine was used in the reaction. The product of Michael-type addition **44** still remained as a major product in the presence of potassium fluoride as a *trans*-isomer.

**Scheme 35:**
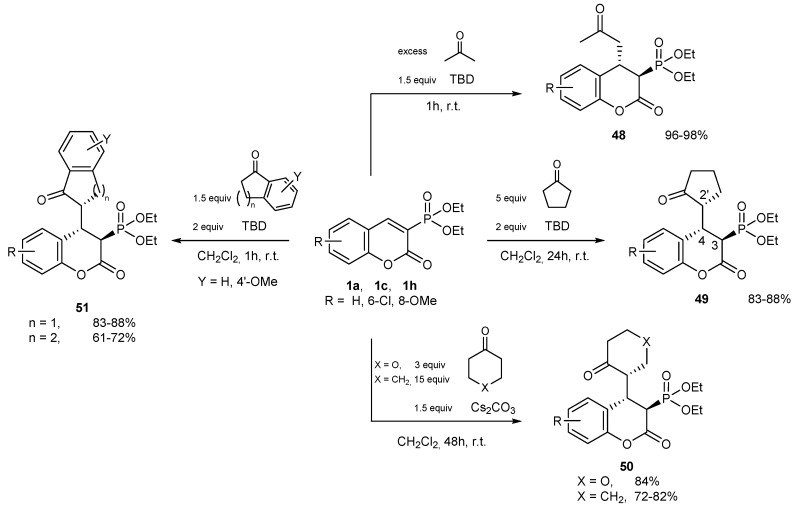
Michael addition of enolizable ketones to 3-diethylphosphonocoumarin **1** using TBD and Cs_2_CO_3_.

A diastereoselective Michael addition of enolizable ketones to 3-diethylphosphonocoumarin **1a**, **1c** and **1h** promoted by 1,5,7-triazabicyclo [4.4.0]dec-5-ene (TBD) was performed in the next work of Krawczyk et al. [59,60] (Scheme 35). Due to the equilibrium of the process, the reactions were performed in the presence of an excess of the used ketone and the TBD base. The electron-withdrawing and electron-donating substituents equally influenced 3-diethylphosphono- coumarins **1c** and **1h** and contributed to their high reactivity. 

Ketones as acetone, cyclopenatanone and 1-indanone have shown high stereoselectivity for the addition products **48**, **49**, **51**. In the case of cyclohexanone, a mixture of unidentified phosphorus-containing products was afforded and the use of cesium carbonate (Cs_2_CO_3_) resolved the problem, producing **50** in very good yields.

**Figure 7:**
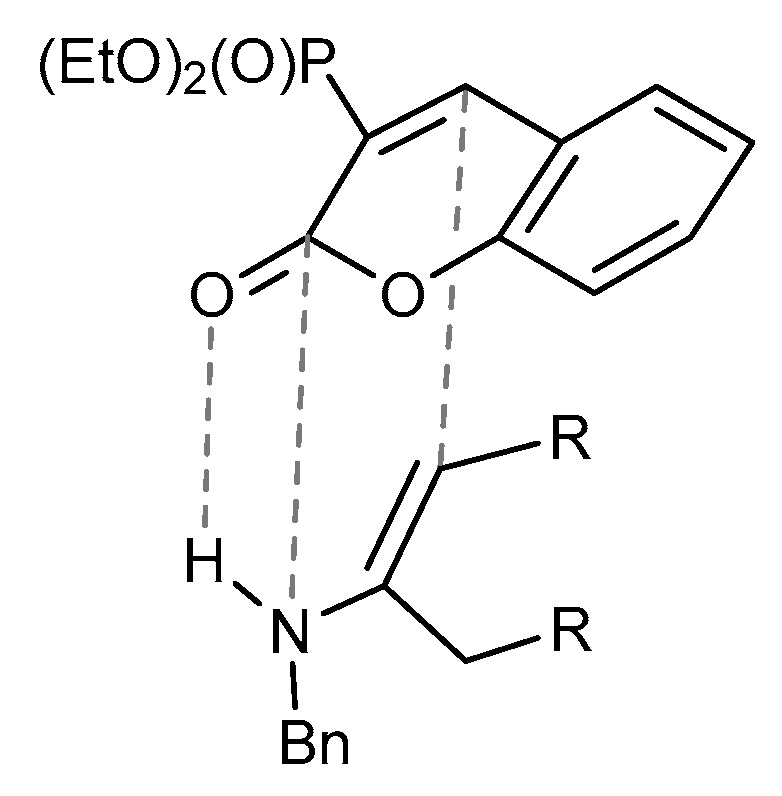
Proposed synclinal transition state formation.

All of the α-phosphono-δ-lactonic products **48**–**51** were isolated as a mixture of C_3_-C_4_ *cis*- and *trans*-isomers with predominant formation of the *trans*-product. A synclinal transition state might be formed when the process was carried out with prochiral ketones. In the described case the Re-face of the enolate approaches the Re-face of the 3-diethylphosphonocoumarin (Figure 7) leading to the formation of a single C_4_-C_2_-*syn* diastereomer (Scheme 35).

**Scheme 36:**
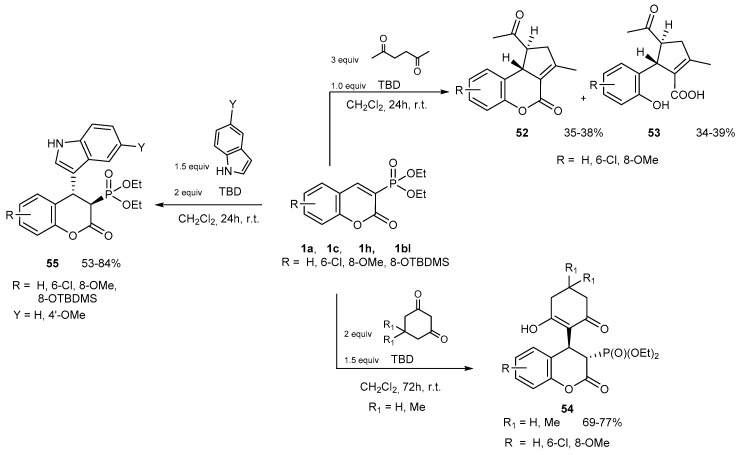
Michael addition of enolizable ketones to 3-phosphonocoumarin **1** using TBD.

In tandem Michael-intermolecular Horner-Wadsworth-Emmons reactions involving 3-diethylphosphonocoumarin **1a** and its substituted analogues **1c** and **1h** (Scheme 36), an alternative CH-acidic compound, 2,5-hexanedione, was applied [61]. The performed reactions were catalyzed by a stoichiometric amount of TBD at room temperature. The quenching of the process resulted in the formation of the *trans*-isomer of product **52** and the corresponding hydroxyacid **53** in almost equimolar amounts. The formation of product **53** was in a result of subsequent hydrolysis catalyzed by the presence of TBD of the formed benzolactone adduct. Modified conditions utilizing a protic solvent (MeOH), were applied to favor the formation of **53** as a sole product. However, the products of type **53** were isolated as their methyl *trans*-cyclopentencarboxylate derivatives.

Then the dione system was changed to cyclohexane-1,3-dione [62] products **54** were observed (Scheme 36) and later used as adducts in a subsequent domino transesterification-cyclodehydration process that resulted in the formation of methyl 2-(diethoxyphosphoryl)-2-(1-oxo-2,3,4,9-tetra- hydro-1*H*-xanthen-9-yl)acetates. Products **54** were isolated as single diastereomers with *anti*-disposition of the bulky groups.

A functionalization of the indole structure was performed by a conjugate addition of substituted indoles to 3-diethylphosphonocoumarins **1a**, **1c**, **1h** and **1bl** [63]. The reaction was carried out in the presence of TBD in CH_2_Cl_2_ at room temperature and products **55** were isolated as a mixture of *cis*- and *trans*-diastereomers, the applied conditions favored the formation of the *trans*-adducts. In the presence of TBD, substituted 3-diethylphosphonocoumarins **1c**, **1h** and **1bl** participated with high efficiency in the Michael-type addition process regardless the presence of electron-withdrawing or electron-donating groups in the benzene moiety. 

## 3. Synthesis and Some Reactions of Alkyl 1,2-benzoxaphosphorin-3-carboxylates 2

### 3.1. Synthesis of Substituted Alkyl 1,2-benzoxaphosphorin-3-carboxylates **2**

#### 3.1.1. Synthetic Protocols Involving Knoevenagel Condensation Reaction

The synthesis, isolation and characterization of the phosphoroheterocyclic analogue of 3-dialkylphosphonocoumarin, the corresponding alkyl 1,2-benzoxaphosphorin-3-carboxylates **2a**–**f** via Knoevenagel lactonization were previously discussed [17,20] in Section 2. 

**Scheme 37:**
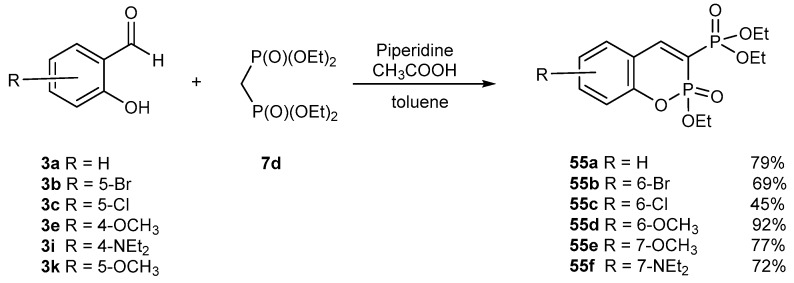
Synthesis of dialkyl 1,2-benzoxaphosphorin-3-phosphonates **55** via Knoevenagel reaction.

A synthetic protocol including the formation of dialkyl 1,2-benzoxaphosphorin-3-phosphonates **55a**–**f** [64] under Knoevenagel reaction conditions (Scheme 37) was performed by using substituted salicylaldehydes **3a**–**c**,**e**,**i**,**k** and tetraethyl methylenebisphosphonate (**7d**). The products **55a**–**f** were isolated in yields from 69 to 92%.

The optimized reaction conditions were elaborated on the reaction of salicylaldehyde (**3a**) and tetraethyl methylenebisphosphonate (**7d**) in the presence of different catalysts—piperidine, piperidine/AcOH, piperidine/ClCH_2_COOH and piperidine acetate/β-alanine. The best results were obtained by slow addition of the used catalyst – piperidine or combination of piperidine/acetic acid. The low activity of the methylenebisphosphonate **7d** as a participating CH-acidic component was the reason for the used excess of salicylaldehyde in comparison with the amount used for the Knoevenagel reactions in the presence of phosphonoacetate **4**.

In the described conditions, the aromatic aldehydes bearing electron-donating groups showed high reactivity toward the condensation reaction, whereas, moderate yields were obtained in the presence of electron-withdrawing groups in the benzene moiety (45–69%).

#### 3.1.2. Synthetic Protocols Including Intermolecular Horner-Wadsworth-Emmons Reaction

**Scheme 38:**
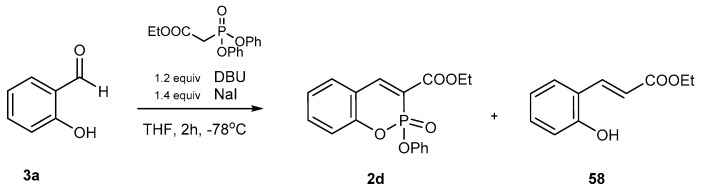
Horner-Wadsworth-Emmons reaction catalyzed by DBU.

Similar conditions were used in the reaction of salicylaldehyde (**3a**) with ethyl diphenylphosphonoacetate in the presence of DBU and different salts (NaI, LiCl, KI, MgBr_2_) as catalytic systems (Scheme 38). A process involving intermolecular Horner-Wadsworth-Emmons reaction instead of Knoevenagel condensation resulted in the formation of ethyl 1,2-oxaphosphorine-3-carboxylate (**2d**). The best results were obtained by applying NaI as a catalyst and the reaction was performed at low temperatures, thus affording the main product **2d** in a yield of 61% [65]. A process involving HWE reaction instead of Knoevenagel condensation was postulated, otherwise the more stable *erythro*-aldol adduct should give the *E*-alkene and therefore the corresponding 3-diethylphosphonocoumarin **1a**. The less stable *threo*-aldol intermediate—the *Z*-alkene, afforded the ethyl 1,2-oxaphosphorine-3-carboxylate **2d** (the intermediates were discussed in Section 2, Scheme 5). A reaction of triethyl phosphonoacetate **4**, salicylaldehyde (**3a**) and DBU under refluxing toluene was carried out to test the proposed transformation path. Neither the 3-dialkylphosphonocoumarin **1a** nor the 1,2-oxaphosphorine **2d** were registered, therefore on the base of the made comparison *erythro*-aldol adduct was accepted as intermediate in the applied conditions. 

In the same conditions, the performed reactions underwent as a Knoevenagel condensation (Scheme 5) in the presence of piperidine or as HWE when DBU was used (Scheme 38). Therefore, the outcome of the transformation depended strongly on the base applied in the reaction.

#### 3.1.3. Synthetic Protocols on the Reaction of Oxaphospholes with Terminal Acetylenes

**Figure 8:**
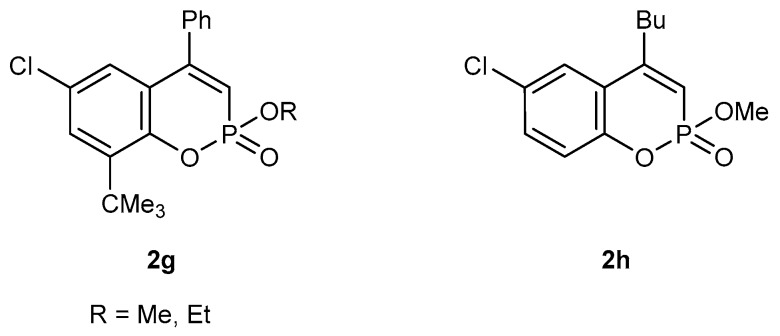
The structures of obtained 1,2-benzophosphorins of type **2**.

**Scheme 39:**
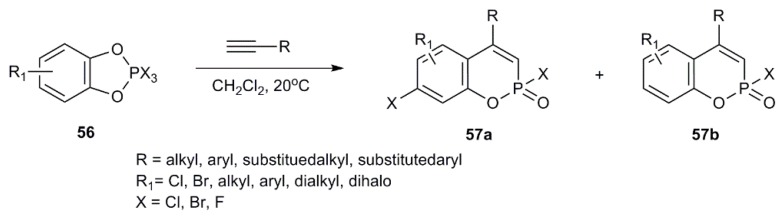
Reactions involving oxaphospholes.

Substituted alkyl 1,2-benzoxaphosphorins of type **2**, Figure 8, were obtained in reaction of 2,2,2-trichlorobenzo-1,3,2-dioxaphosphole (**56**) with phenyl- and alkylacetylenes [66,67] (Scheme 39). The obtained 2,6-dichlorobezno[e]-1,2-oxaphosphinine-2-oxides **57** were converted to esters in subsequent reactions with methanol, ethanol or triethyl orthoformate. The extensive research of Mironov and coworkers was summarized in a review paper [68] presenting the synthesis of numerous halophosphorinines.

The described method combined the properties of P,P,P-trihalobenzodioxaphospholes derived from 1,2-benzoquinones with a phosphorus trihalide. It is known that compounds like P(V) halides interact with C≡C bond to form P-C bonda. During this transformation, the phosphorus atom changed from a five- to four-coordinated state. Using the properties of the dioxaphospholes the reactions with terminal aryl- and alkylacetylenes were performed affording series of 2,6-dichlorobezno[e]-1,2-oxaphosphinine-2-oxides (**57**) (Scheme 39). The regioselectivity of the reaction depended not only on the substituents on the phosphorus atom but also in the aromatic moiety of the P,P,P-trihalobenzodioxaphospholes **56** as well as substituents on the alkynyl component. In all reaction conditions, a halogenation of the aromatic fragment from the released halogen of the phosphole occurred. In most of the performed reactions, a mixture of 2-halo-1,2-phosphorinines **57a** and **57b** was produced and the regiochemistry of the process is still not identified. Several reaction mechanisms were proposed based on the obtained results by different researchers utilizing variously substituted starting compounds. 

In subsequent papers, the electronic effect of donor and acceptor substituents at the arylacetylenes on the reaction rate was tested [69,70,71,72]. With the enhancement of the electron-donor properties of the substituent, the reaction rate increased. Moreover, donor and acceptor effect of the substituents at fourth position in the 1,2-oxaphosphinine ring influenced the electrophilicity of the phosphorus atom.

The 2-halo-1,2-phosphorinine adducts **57a**,**b** were converted into acids, amides and salts with different counterions with the sole purpose to apply them as promising additives for polymers, ligands for metal complex catalysis and etc.

#### 3.1.4. Synthetic Protocols on A Reaction of Dialkyl Benzylphosphonates with Methyl Salicylate

**Scheme 40:**
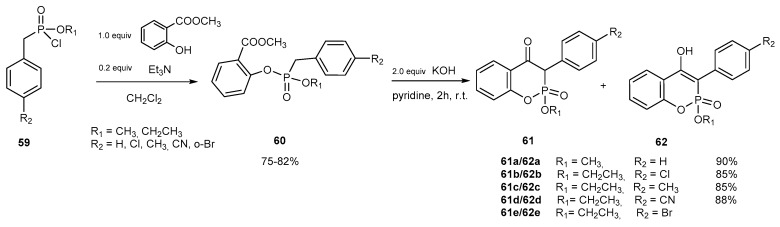
Multistep procedure for obtaining P-heterocyclic products.

A multistep synthesis of 3-aryl 4-hydroxy-1,2-oxaphosphorines **62** and 3-arylphospha- chroman-2,4-diones **61** was presented by Fu and coworkers [73,74]. It began with the preparation of dialkyl benzylphosphonates in yields of 80–85%. The obtained benzylphosphonates were converted to the more reactive halogenides **59** and in a subsequent reaction with methyl salicylate afforded the corresponding phosphonates **60** in very good yields (75–82%). The third step included a P-heterocyclic compound formation in the presence of KOH in dry pyridine. The product of the cyclization process was a mixture of two tautomers with predominate keto vs. enol-form, compounds **61** and **62** (Scheme 40).

**Figure 9:**
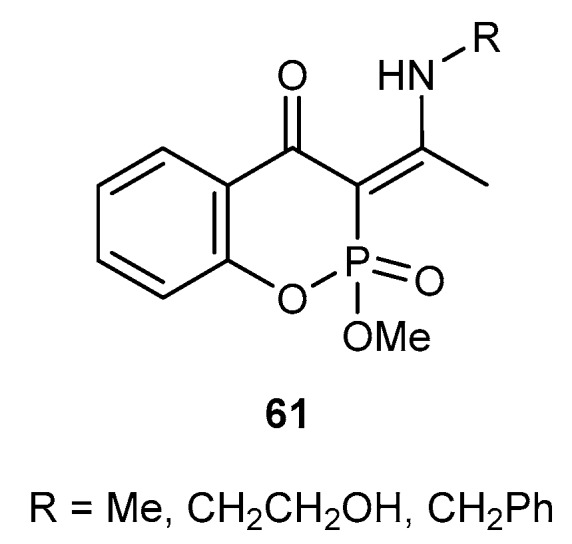
Another example of compounds **61**.

Compounds of type **61** (Figure 9) were obtained by a rearrangement reaction and their alkylating effect and cytotoxicity was tested on some human leukemia cell lines HL-60 and NALM-6 [41]. They have shown high alkylating activity in respect to both cell lines but were less active then 3-dialkylphosphonocoumarins **1l**–**p**. 

#### 3.1.5. Synthetic Protocols on for Reactions of 2-Ethoxyvinylphosphonic Dichloride with Substituted Phenols

**Scheme 41:**
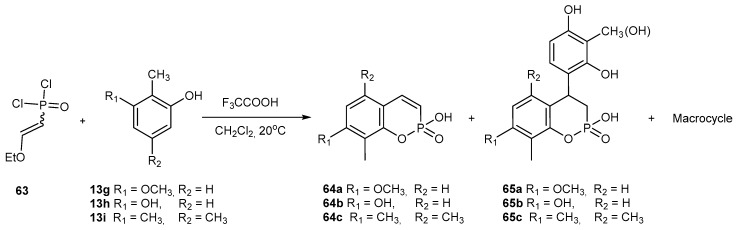
Condensation of a phosphorus-bearing component with substituted phenols.

The 1,2-oxaphosphorines **64a**–**c** and **65a**–**c** were isolated in low yields during the synthesis of macrocyclic P-containing phenols [75]. The method utilized 2-ethoxyvinylphosphonic dichloride (**63**) and resorcinols **13g**–**i** in ratio 1:2 as starting materials and trifluoroacetic acid as catalyst (Scheme 41). The ratio between the 1,2-oxaphosphorine adducts **64a** and the macrocyclic compound was found to be 1:1, whereas, compound **65a** was isolated in a yield of only 5%. In a subsequent paper [76] the reaction was carried out in dioxane as a solvent at 60 °C thus resulting in reduction of the yield of 1,2-oxaphosphorine **64a**. One of the possible explanations for the observed reaction course was the formation of the halogenide of oxaphosphorine as the main intermediate in the synthesis of the macrocycles and the corresponding oxaphosphorine **64a** was formed as a result of a parallel interaction. Therefore, a modified procedure was published in 2015 representing [77] a condensation method for preparation only of 1,2-oxaphosphorines **64c** and **65b**,**c** in toluene as a solvent media under reflux in yields of 75–85%. 

#### 3.1.6. Synthetic Protocols for Gold-Catalyzed Hydroarylation of Aryl Alkynylphosphonates

**Scheme 42:**
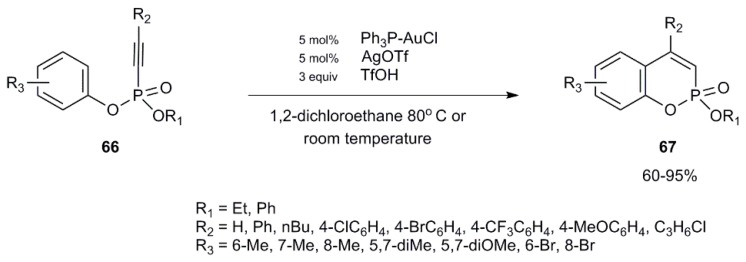
Gold-catalyzed hydroarylation of aryl alkynylphosphonates.

An alternative approach [78] for the preparation of the target 1,2-oxaphosphorines involved an intramolecular hydroarylation of a series of substituted aryl alkynylphosphonates in the presence of gold catalysts, silver salts and a protic acid. Different gold compounds were applied for optimizing the reaction conditions. The highest results (yield of 84%) were observed when a combination of Ph_3_P-AuCl, AgOTf and TfOH was utilized for the activation of the triple bond (Scheme 42). Conditions where the reaction mixture was heated at 80 °C or the process was performed at room temperature, were both applicable. However, the best results for the hydroarylation were obtained at room temperature. Substituted diphenyl and ethyl phenyl alkynylphosphonates **66** afforded a broad range of 1,2-oxaphosphorine adducts **67** in yields of 60–95%. Elaborating on the mechanism of the reaction, alkynylphosphonates with substituents on the phenyl ring attached to oxygen was also examined. A relationship between the steric effect of the group **R^2^** and the obtained yields for 1,2-benzoxaphosphorines **67** was noted, when the hindrance effect of the group increased the yields of **67** decreased from 94 (R^2^ = Ph) to 60% (R^2^ = *n*-Bu or C_3_H_6_Cl).

#### 3.1.7. Synthetic Protocols for Pd-Catalyzed Intramolecular Cross-Coupling Reactions of Ethyl 2-(aryl)arylphosphonates

**Scheme 43:**
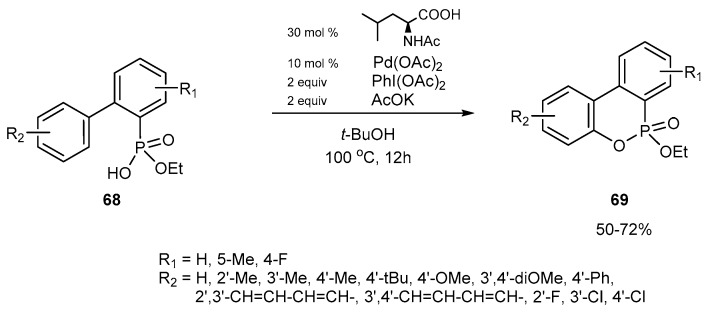
Intramolecular Pd-catalyzed cyclization reaction.

Intramolecular Pd-catalyzed oxidative-cyclization reaction for the formation of 1,2-oxaphosphorine derivatives **69** involving ethyl 2-(phenyl)phenylphosphonate and substituted alkyl 2-(aryl)arylphosphonates **68** was presented by Lee et al. [79]. The best reaction conditions included a combination of 10 mol % Pd(OAc)_2_ and two equiv. PhI(OAc)_2_ with N-acetyl-L-leucine as an additional ligand for the Pd(II)–Pd(IV) conversion. Substituted 1,2-oxaphosphorines of type **69** were isolated in yields of 50–72%, Scheme 43. 

**Scheme 44:**
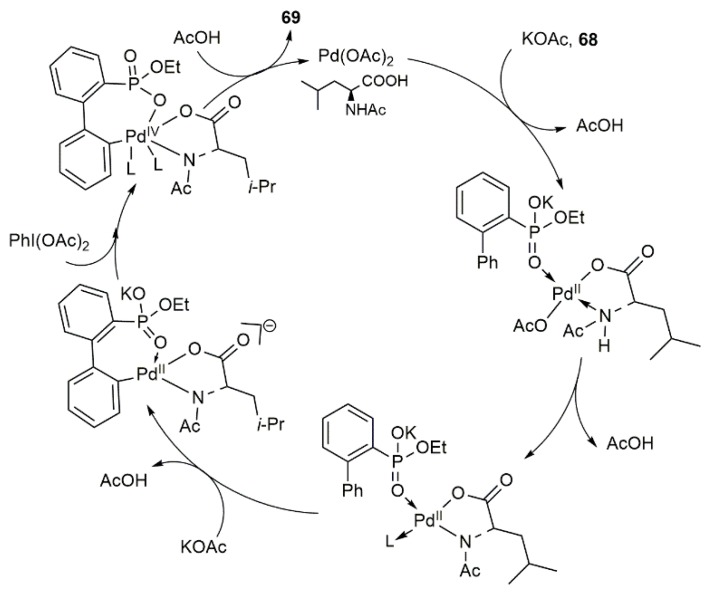
Proposed mechanism of the intramolecular Pd-catalyzed cyclization reaction.

The study on the mechanism of the cyclization showed that the C-O bond formation was the rate-determining step in which Pd(II)/Pd(IV) catalytic cycle might be involved (Scheme 44).

### 3.2. Chemical Reactions of Alkyl 1,2-benzoxaphosphorin-3-carboxylates **2**

#### 3.2.1. Reactions Resulting in the Formation of 4-*O*-substituted 1,2-benzoxaphosphorines

**Scheme 45:**
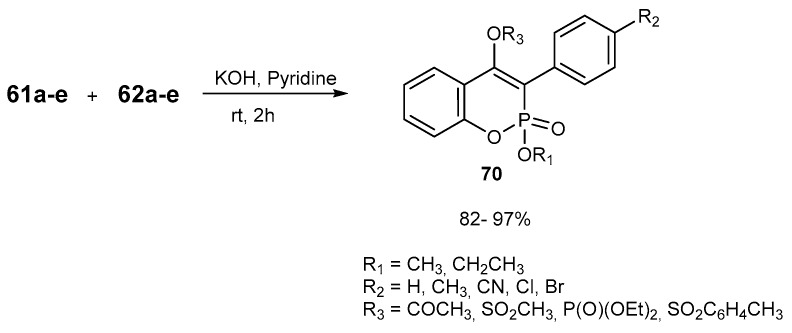
Formation of 1,2-benzoxaphosphorines **70**.

3-aryl-4-hydroxy-1,2-oxaphosphorine compounds **61a**–**e** and 3-arylphosphachroman-2,4- diones **62a**–**e** were used [73] in reactions with acetic anhydride, methylsulfonyl chloride, diethyl phosphorochloridate and *p*-toluenesulfonyl chloridate in the presence of K_2_CO_3_ in acetone (Scheme 45). The corresponding 4-O-substituted 1,2-benzoxaphosphorines **70** were isolated in yields of 82–97%.

The obtained compounds **61a**–**e**, **62a**–**e** and **70** were tested as inhibitors against the enzyme SHP-1, a member of the protein tyrosine phosphatase (PTP) family responsible for the regulating of numerous cellular processes. Irregular functioning of this protein family could lead to cellular dysfunction and various diseases, thus, PTP-inhibitors could provide potential therapeutic agents against such abnormalities. Most of compounds **70** exhibited certain membrane-permeable PTP-inhibitor activity, however, only the 1,2-benzoxaphosphorines bearing R_3_ = diethoxyphosphoryl group, R_2_ = Cl and R_1_ = Et indicated better inhibition properties.

**Scheme 46:**
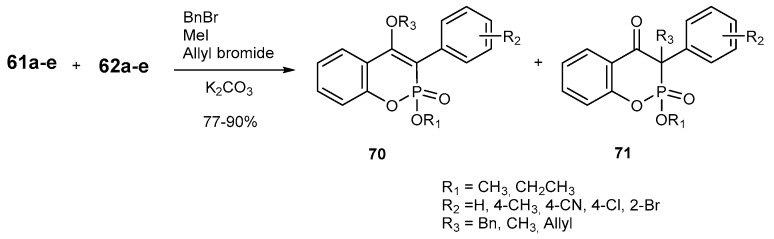
Alkylation reaction to products **70** and **71**.

The same mixture of tauromers **61a**–**e** and **62a**–**e** was implied [74] in an alkylation reaction with series of alkyl halides. Products of C- and O-alkylation **70** and **71** were isolated in total yields of 77–90% (Scheme 46). The ratio of the two products depended on the steric effect of the R_2_-substituent on the benzene ring in third position of the oxaphosphorin systems. Substituents at *ortho*-position favored the formation of O-alkylated product. The steric hindrance effect of the alkyl halide was also apparent. In the case of CH_3_I the ratio was shifted to the C-alkylated product whereas with bulky alkyl groups the proportion of the two products was almost equal.

#### 3.2.2. Participation in Coupling Reactions

**Scheme 47:**
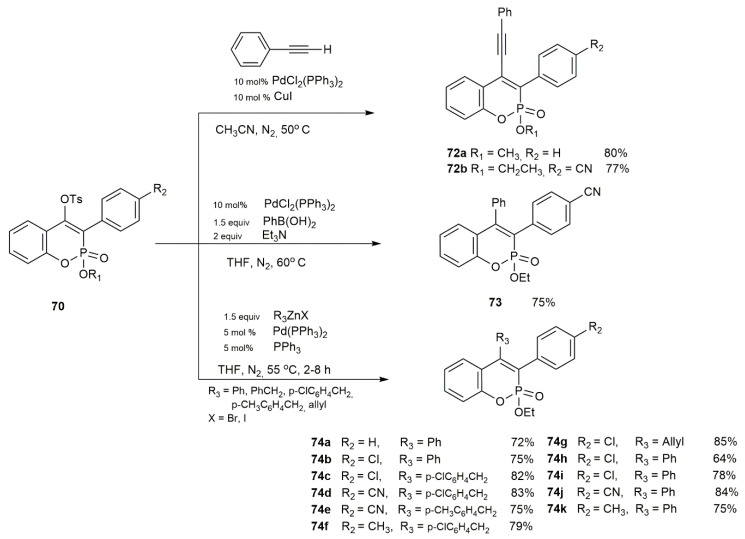
Conditions and results for the coupling reactions.

In order to construct a variety of complex structures, 4-tosylphosphacoumarins of type **70** were applied [73] in Sonogashira and Suzuki coupling reactions in the presence of 10% mol PdCl_2_(PPh_3_)_2_ and CuI or Et_3_N as an additive affording the formation of 4-aryl- and 4-alkynylderivatives **72** and **73** (Scheme 47). 

The same research group [80], accomplished Negishi cross-coupling reaction of 4-tosylphosphacoumarins **70** in the presence of a catalytic system including 5 mol % Pd(PPh_3_)_4_ and 5 mol % PPh_3_ (Scheme 47). The expected products **74a**–**k** were isolated in 64–85% yield. 

**Scheme 48:**
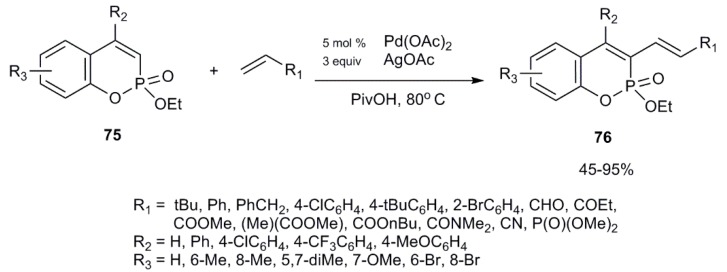
Palladium-catalyzed Heck reaction.

Alkenylation [81] of 1,2-oxaphosphorines of type **75**, Scheme 48, was carried out in Heck reaction conditions in the presence of Pd-catalyst. The corresponding 1,2-benzoxaphosphorines **76** were isolated in yields of 45–95%. The reaction efficiency was not dependable on the used substituents on the aryl or the styrene moieties. 

**Scheme 49:**
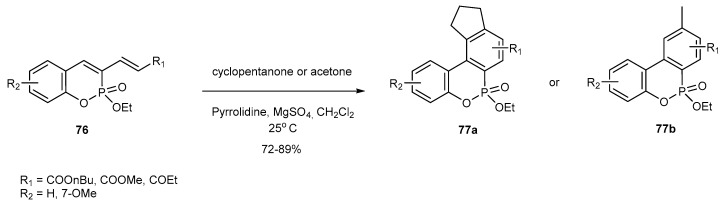
Accomplishing a Diels-Alder reaction with compounds **76**.

Furthermore, compounds **76** were used as diene systems and underwent inverse Diels-Alder reactions with enamines (Scheme 49). Subsequent 1,2-elimination/dehydrogenation afforded the fluorescent 1,2-benzoxaphosphorine structures of type **77a**,**b** in very good yields (72–98%).

## 4. Reactions of [2+2] and [3+2] Cycloadditions of Dialkyl 2-oxo-2*H*-1-benzopyran-3-phosphonates and Alkyl 1,2-benzoxaphosphorin-3-carboxylates

### 4.1. [2+2] Cycloaddition Reactions

**Figure 10:**
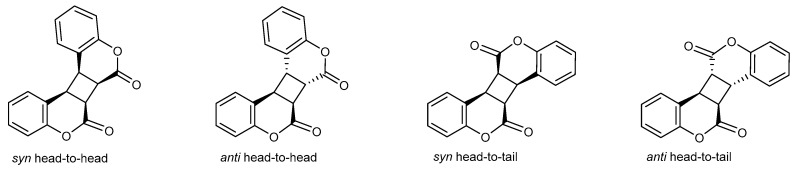
Cyclized stereoisomers.

The photochemical dimerization of coumarins is frequently used in organic synthesis. The possible stereoisomers of the coumarin structure are given in Figure 10, representing different C_3_=C_4_ double bond cyclization products. 

**Scheme 50:**
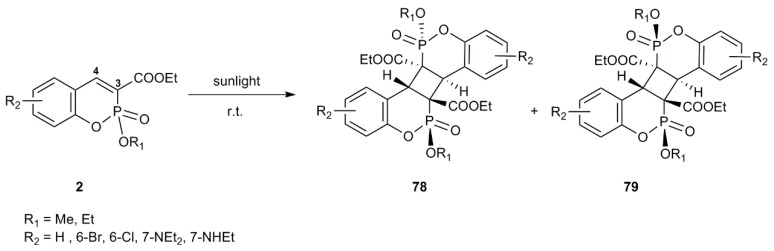
[2+2] Cycloaddition products.

Alkyl 1,2-benzoxaphosphorine-3-carboxylates have shown similar behavior [82], leading to the formation of only anti-head-to-tail dimers **78** and **79** (Scheme 50). The reaction was accomplished in a series of solvents from which protic polar solvents accelerate the dimerization process under sunlight irradiation. Electron-withdrawing substituents in the benzene ring of **2** stabilized the intermediates formed during cyclization and enhanced the yield of the stereoisomers, whereas electron-donating groups such as diethylamino did not favor the performed interaction.

Quantum-chemical calculations on the mechanism of the dimerization proposed an asynchronous [2+2] reaction with dominant head-to-tail regioisomers due to the formation of diradicals or dipolar intermediate through C_3_-C_4′_ interaction (Scheme 50).

The different chemical behavior of the two isomers **1** and **2** toward [2+2] cycloaddition reactions was illustrated by their ability to form the corresponding dimers [20]. While 1,2-benzoxaphosphorines **2** easily gave the head-to-tail adducts, 3-diethylphosphonocoumarins **1** have shown lack of reactivity in the described transformation. This observation was due to the more aromatic characteristic of the C3=C4 double bond in the lactone ring of the phosphonocoumarin moiety. 

### 4.2. [3+2] Cycloaddition Reactions

**Scheme 51:**
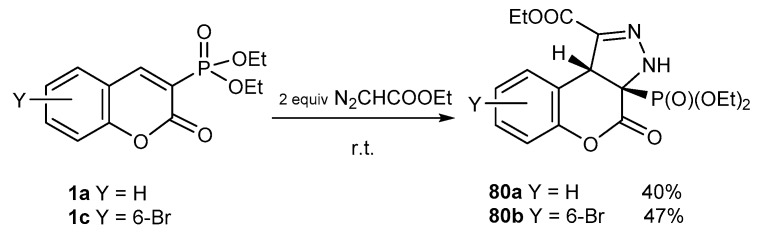
[3+2] Cycloaddition reaction of 3-diethylphosphonocoumarins **1a** and **1c**.

Pyrazoline derivatives of 3-diethylphosphonocoumarin **80a**,**b**, 1,2-benzoxa- phosphorine-3-carboxylates and 1,2-benzoxaphosphorin-3-phosphonates **81** were prepared [64] in reactions with ethyl diazoacetate. The solvent mixture for the three coumarins was different based on their solubility and the solubility of the products. Benzene and chloroform were preferred as solvents in the case of coumarins **1a** and **1c** and pyrazolines **80a**,**b** were isolated in moderate yields (Scheme 51).

**Scheme 52:**
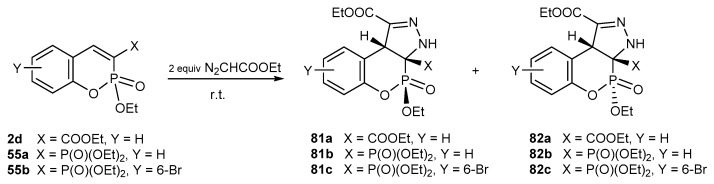
[3+2] Cycloaddition reaction of 1,2-benzoxaphosphorines **2d** and **55a**,**b**.

In general, 1,2-benzoxaphosphorines participate in the cycloaddition reaction in a short time giving higher yields due to the activated and more isolated double bond in the oxaphosphorine ring (Scheme 52). The outcome from the reaction of 1,2-benzoxaphosphorine **2d** was a mixture of two epimeric pyrazolines **81a**–**c** and **82a**–**c** in different ratios. The ratio was influenced by the solvent polarity and the desired cycloaddition products were isolated in good to excellent overall yields ranging from 70% (60 days; benzene/*n*-hexane; ratio **81**:**82**—1:0.25) to 95% (30 days; CH_2_Cl_2_/*n*-hexane; ratio **81**:**82**—1:0.14). The ether/*n*-hexane solvent mixture occurred to be the best condition for 1,2-benzoxaphosphorin-3-phosphonates **55a**–**b** affording target products **81b**,**c**/**82b**,**c** in overall yield of 76% and 70%, respectively. In all the cases the major isomer was **81a**–**c** with *cis*-disposition of P=O group and substituent at 3a-position (COOEt, P(O)(OEt)_2_).

## 5. Conclusions

The interest towards dialkyl 2-oxo-2*H*-1-benzopyran-3-phosphonates and alkyl 1,2-benzoxaphosphorin-3-carboxylates as precursors for biologically active compounds allowed us to summarize the scientific research on their synthesis and participation in conjugate addition reactions. In the beginning the methods for preparation of 3-dialkylphosphonocoumarins were discussed, where Knoevenagel condensation reactione, application of phosphoryl ketenimines or vinylphosphonates, phosphorylation of coumarins, three-component and tandem coupling reactions or rearrangement processes, etc. were presented. The electronic effect of the substituents on the used substrates as well as the applied reaction conditions were accounted for by presenting the mechanisms, where they were given, and the corresponding results from the studies were discussed. The comparisons between the developed methods highlighted the advantages and drawbacks in the selected research papers. The protocols for the synthesis of alkyl 1,2-benzoxaphosphorins were also summarized, where reactions of oxaphospholes with terminal acetylenes, dialkyl benzylphosphonates with methyl salicylate, etc. were analyzed.

The chemical behavior of dialkyl 2-oxo-2*H*-1-benzopyran-3-phosphonates in conjugate addition reactions with different nucleophilic reagents—hydride ion, organometallic and CH-acidic compounds—as well as reactions of [2+2] and [3+2] cycloaddition were reviewed in details.

## Figures and Tables

**Table 1:** Reaction conditions for the preparation of **1a**–**c,j** and **2b**–**f** using different catalysts.

R^1^	X	R	Method	Overall Yield	1 ^a^	2 ^a^
Me	COOEt	H	A	51	33	11
			A^1^	56	27	21
			B	20	20	0
Et	COOEt	H	A	87	70	9
			A^1^	98	84	14
			B	50	50	0
			C	74	66	8
Et	COOSi(Me)_3_	H	A	59	47	8
			C	28	20	2
			D	73	57	9
Et	CN	H	A	69	45	24
			A^1^	52	17	35
			B	49	49	0
			C	55	47	8
			D	60	42	18
Et	COOEt	6-Br	A	98	77	19
			B	81	81	0
			C	50	40	4
Et	COOEt	6-Cl	A	78	65	9
			B	71	71	0
			C	67	41	20
Et	COOEt	7-NEt_2_	A	81	64	17
			B	3	3	0
			C	86	71	15

Method A: Toluene/piperidine/Dean-Stark trap; Method A^1^: Toluene/piperidine/Dean-Stark short distance trap; Method B: THF/TiCl_4_/pyridine; Method C: Toluene/β-alanine/piperidine acetate/Dean-Stark short path trap; Method D: Toluene/piperidine/mol. siev. 4A, reflux. ^a^ Isolated yields.

**Table 2:** Results from the reactions of 2-diethoxyphosphorylacrylates **12** with substituted phenols or naphthols **13** in the presence of strong acids.

1	R_1_	R_2_	R_3_	R_4_	R_5_	Reaction Time [days]	Yields	Ref
**1q**	H	OMe	H	OMe	H	6d	88%	[23,24,25]
**1e**	H	H	H	OMe	H	10d	81%	[24,25]
**1r**	H	H	-CH_2_OCH_2_-		H	60d	73%	[24,25]
**1f**	H	-CH=CH-CH=CH-		H	H	10d	88%	[24,25]
**1s**	H	-CH=C(OH)-CH=CH-		H	H	18d	95%	[24,25]
**1t**	H	H	H	-CH=CH-HC=CH-		14d	9%	[24]
**1u**	Me	H	H	OMe	H	3d	81%	[26,27]
**1v**	Me	OMe	H	OMe	H	3d	69%	[26]
**1w**	Me	H	H	-CH=CH-HC=CH-		3d	60%	[26]
**1x**	Et	H	H	OMe	H	3d	59%	[26]
**1y**	Et	OMe	H	OMe	H	3d	47%	[26]
**1z**	Et	H	H	-CH=CH-HC=CH-		3d	26%	[26]
**1aa**	n-Bu	H	H	OMe	H	3d	38%	[26]
**1ab**	n-Bu	OMe	H	OMe	H	3d	31%	[26]
**1ac**	Ph	H	H	OMe	H	3d	85%	[26]
**1ad**	Ph	OMe	H	OMe	H	3d	70%	[26]

**Table 3:** Comparison between the results for reaction of **1a** with organomagnesium reagents under thermal and ultrasonic initiation.

Substituent R_1_	Reflux		US	
	Reaction Time [min]	Yield [%]	Reaction Time [min]	Yield [%]
Et	50	64	10	89
*n*-Pr	60	77	30	94
*i*-Pr	80	69	50	74
PhCH_2_	125	38	90	53
CH_2_COO*t*-Bu	75	78	30	95
